# Heterogeneity in disease resistance and the impact of antibiotics in the US

**DOI:** 10.1016/j.ehb.2022.101155

**Published:** 2022-07-27

**Authors:** C. Justin Cook, Jason M. Fletcher

**Affiliations:** a University of California-Merced, United States; b University of Wisconsin-Madison, United States

**Keywords:** Difference-in-differences, Human Capital, Infectious disease, Genetic Resistance, Genetic Diversity, Antibiotics

## Abstract

We hypothesize that the impact of antibiotics is moderated by a population’s inherent (genetic) resistance to infectious disease. Using the introduction of sulfa drugs in 1937, we show that US states that are more genetically susceptible to infectious disease saw larger declines in their bacterial mortality rates following the introduction of sulfa drugs in 1937. This suggests area-level genetic endowments of disease resistance and the discovery of medical technologies have acted as substitutes in determining levels of health across the US. We also document immediate effects of sulfa drug exposure to the age of the workforce and cumulative effects on educational attainment for cohorts exposed to sulfa drugs in early life.

## 1. Introduction

At the beginning of the 20th century, deaths per 1000 live births in the United States within the first month and first year of life were nearly 50 and 150, respectively. One century later, early life mortality has been substantially reduced to 4.6 deaths in the first month and 6.9 deaths in the first year.^1^ A key determinant of this decline is improvements in environmental factors such as nutrition and sanitation, access to effective care, and innovations in medical technologies and information (Armstrong et al., 1999; CDC, 1999). For example, water filtration and chlorination, which were introduced to the US in the early 20th century, reduced total mortality by 13 %, infant mortality by 46 %, and childhood mortality by 50 % (Cutler and Miller 2005). While these historical examples suggest the possibility that extending these improvements to new and underserved populations could likewise result in large scale increases in population health, it is also possible that extending these improvements could be less effective than predicted due to heterogeneity in effects. One source of this heterogeneity is the extent to which new medications and other innovations substitute for, or complement, existing characteristics in the population and their environments. Specifically, the long histories of interactions around the world between people and bacteria have shaped both the people and the bacteria, leading to “natural” resistances in some populations and a greater susceptibility in others that may moderate the usefulness of medications.

This paper explores the idea of interaction between two principal classes of determinants—that medical technologies may interplay with inherent population characteristics. By incorporating population genetics ideas into a macroeconomic health analysis, we pursue the hypothesis that medical innovations related to infectious disease may have systematically differential effectiveness that depends on the population under study, with the implication that impact estimates of new medications in one population may not extrapolate to others.^2^ To the best of our knowledge, we are the first to explore the interaction between inherent immune differences and an exogenous medical shock.

The focus of the analysis is on bacterial infectious diseases in the United States, where we exploit a genetically determined population resistance phenomenon akin to herd immunity that preceded modern medicine. A hypothesis put forward by population geneticists and others ties differences in population-level resistance to the large amount of diversity in the set of genes associated with the recognition and disposal of foreign pathogens, the human leukocyte antigen (HLA) system (Cagliani and Sironi, 2013; Prugnolle et al., 2005; Sabeti et al., 2006). Genetic diversity within the HLA system provides resistance to populations by slowing the spread of infectious pathogens (Meyer et al., 2017; Penn et al., 2002; Spurgin and Richardson, 2010). Population level HLA variation has been shown to be strongly associated with cross-country health outcomes in years prior to, but not after, the mid-20th century health innovations, consistent with the possibility of substitution effects between medical innovation and population genetics (Cook, 2015).

Our analysis examines heterogeneity in mortality declines following the discovery and widespread usage of antibiotics in 1937 in the US (Jayachandran et al., 2010). We show that the beneficial effect of antibiotics was not the same for all populations: more susceptible groups (i. e., more HLA similarity) benefit *more* from the introduction of antibiotics, while more resistant groups (i.e., more HLA diversity) benefit less.

**Fig. 1.**
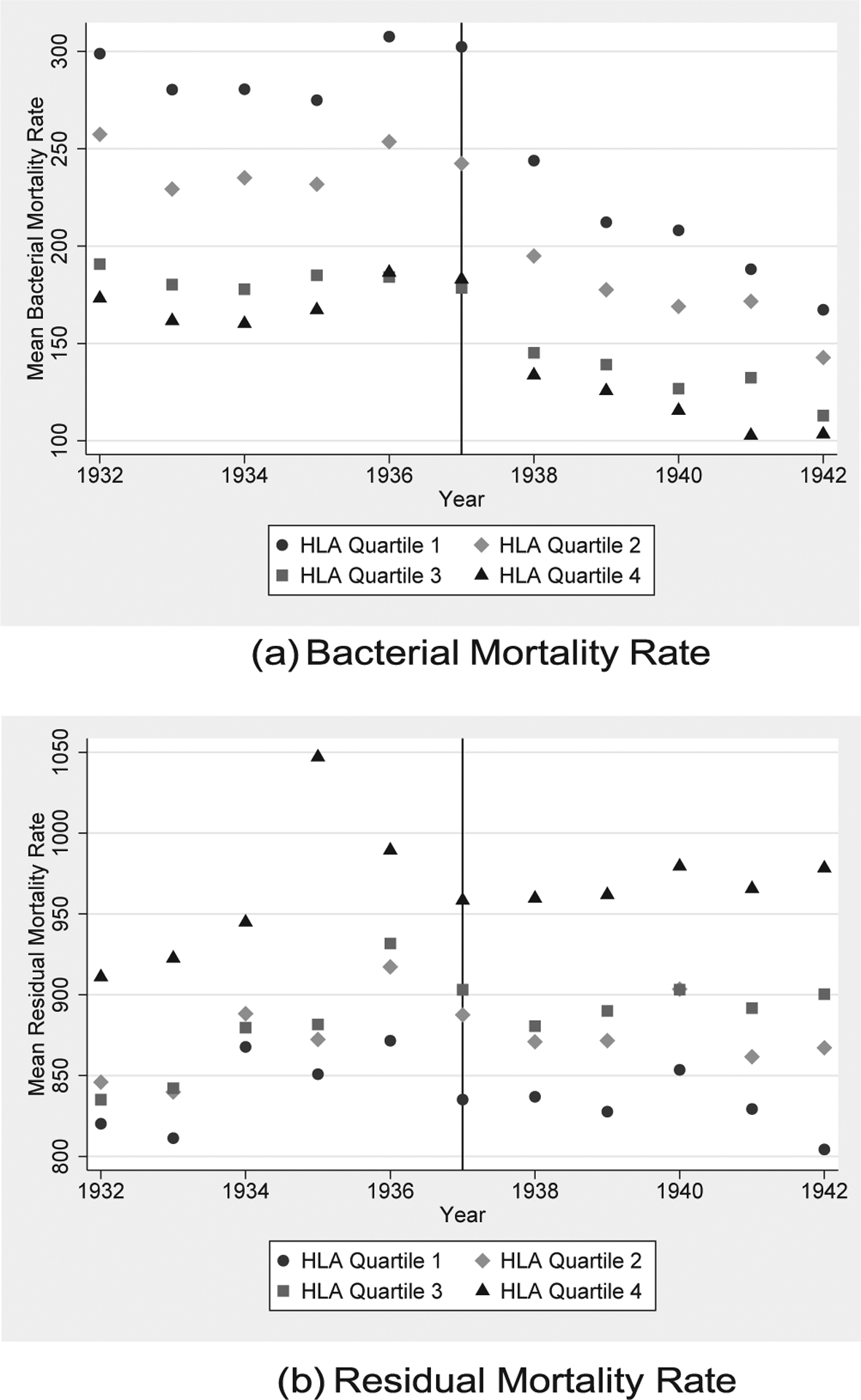
Mean Mortality Rate by HLA Quartiles. Notes: Sub-figure (a) plots the yearly average of bacterial mortality by quartiles of HLA susceptibility for a panel of states 5 years before and after the introduction of sulfa drugs in 1937. A clear reduction is seen following 1937, and a general convergence in bacterial mortality is seen in the following years. Sub-figure (b) plots the yearly average of all cause mortality rate less bacterial causes (i.e., residual mortality) for HLA quartiles. No clear post-1937 change is seen.

We test our hypothesis in a US state panel centered around the 1937 introduction date of sulfa drugs. In so doing, we construct a state-level measure of HLA genetic homozygosity, or similarity, for a representative population prior to the treatment year of 1937. Preliminary evidence of the heterogeneous decline in bacterial mortality rates is presented in Fig. 1a, which plots mean bacterial mortality rate for quartiles by state genetic susceptibility (i.e., HLA similarity). As shown, more susceptible states (quartile 1) have initially higher relative levels of bacterial mortality in periods prior to 1937. But after 1937, all states are shown to have sharp declines in mortality that are visually larger for more susceptible states. It is this more rapid decline tied to a state population’s genetic susceptibility that we estimate. In contrast, Fig. 1b shows that the mortality rate from all other causes is not related to the 1937 intervention and is inversely related to our measure of genetic susceptibility.

### 1.1. Background of HLA diversity: functions and hypothesized causes for population variation

Our mechanism of genetic susceptibility (resistance) is a measure of genetic similarity (diversity) within the set of genes comprising the HLA system. The HLA (human leukocyte antigen) system is associated with the creation of proteins that recognize and are responsible for removing foreign cells from the body. This system of genes is hypothesized to have undergone recent selection (Sabeti et al., 2006), and it is further hypothesized this selection is not for the uniformity of variants that provide a particular benefit; rather the selection of the HLA system is one for *diversity*—the set of genes comprising the HLA system is one of the most diverse regions in the human genome (Cagliani and Sironi, 2013; Jeffery and Bangham, 2000; Meyer et al., 2017). This genetic diversity within the HLA system is hypothesized to be a broad defense that is not strictly tied to a particular infectious pathogen.

HLA diversity is hypothesized to provide *population-level* resistance by increasing the variety of immune responses within the population (Cagliani and Sironi, 2013; Meyer et al., 2017; Penn et al., 2002). The measles virus provides an illustrative example: Obtaining the virus from a (genetically similar) relative increases the likelihood of death from the virus twofold compared to obtaining the virus from a (genetically distant) unrelated individual (Garenne and Aaby, 1990). For individuals within the population, rare variants (corresponding to rare immune response) are favorable, in that pathogens are likely to adapt and overcome common genetic responses. Having a rare immune response would therefore be beneficial; hence, the rare variant would increase in frequency as the population encounters and is shaped by the particular pathogen.^3^

The roots of population differences in the level of genetic diversity within the HLA system are tied to the out-of-Africa migration and the Neolithic Revolution.^4^ Serial founder effects^5^ from the migration out of East Africa are associated with overall genetic diversity of a population, including the level of genetic diversity within the HLA system, but outof-Africa migration routes only explain 17–39 % of the variance in HLA diversity compared to 85 % for overall genetic diversity (Ashraf and Galor, 2013; Prugnolle et al., 2005; Qutob et al., 2012; Ramachandran et al., 2005). Additional variation in HLA heterozygosity is tied to the differential timing and composition of the transition to agriculture. Due to differences in the timing of agriculture and the availability of domesticate animals, societies have had historically different exposure to infectious disease (Wolfe et al., 2007). The presence of domesticate animals and the large, dense, and sedentary populations that could only be achieved after agriculture provided the basis for an epidemiological transition that led to a large increase in the number of infectious diseases (Cook, 2015; Crosby, 1986; Diamond, 1998). We take these building blocks of HLA variation and then use the US as a case study to examine interactions between deep, historical variation in HLA and a new medical innovation against infection disease; since nearly all occupants of the US around 1930 were not indigenous, recent pathogen exposure had no time to shape HLA variation, providing an opportunity to examine a novel (population) genetic-by-environment interaction.

## 2. Data and empirical methodology

### 2.1. State-level HLA homozygosity and heterozygosity

Our analysis will be at the state-level within the US, which allows us to avoid country-level confounders that are likely to bias estimations as well as providing quality improvements in many of the health and economic variables used. Our key measure will focus on genetic susceptibility to pathogens, or HLA homozygosity. To create this measure, we start with its complement, HLA heterozygosity. As discussed in greater detail in Cook (2015), the measure of HLA diversity is derived from the Allele Frequency Database, or ALFRED (Kidd et al., 2003). ALFRED contains a wide array of genetic data for approximately 50 anthropologically defined ethnicities, covering all continents in which humans live. Our focus is solely on genetic variants within the HLA System. The HLA system is a collection of 239 genes located on the sixth chromosome (Shiina et al., 2004). From ALFRED, we obtain data for 156 different single nucleotide polymorphisms, or SNPs, for each of 51 distinct ethnicities. Group-level frequencies of these genetic variants, or alleles, are then used to calculate the measure of genetic diversity specific to immune function.

Following Ashraf and Galor (2013), we measure genetic diversity by expected heterozygosity, which is the probability that two individuals differ in their genetic variant at a locus (or SNP in our case). Formally, expected heterozygosity is defined as:

(1) $$H_{exp}=1-\frac{1}{m}\sum_{l=1}^{m}\sum_{i=1}^{k_{l}}p_{i}^{2}$$

where *p*_*i*_ represents the fraction of allele *i* within each population (ethnicity for our case), and expected heterozygosity is found by the average across the *m* loci (the 156 SNPs). To better represent susceptibility and the differential response associated with introduction of sulfa drugs, we consider expected *homozygosity* within the HLA system, which is simply one minus heterozygosity, making the two measures interchangeable (Ashraf and Galor, 2013).

We use data from the 1980, 1990, and 2000 US Census’s 5 % state sample, which is a 1-in-20 random sample of the US population (Ruggles et al., 2017). All 5 % samples include a measure of self-reported ancestry, which is first available in the 1980 sub-sample. We then match this ancestry to either a specific ethnicity or country for which we have prior measurement of HLA heterozygosity.^6^

This measure constructed from the 1980/1990/2000 Census ancestry categories, however, would capture periods that are after the proposed intervention data of 1937. Therefore, to account for the pre-period level of genetic susceptibility, use reported state of birth and age to construct a measure of state-level HLA homozygosity representative for pre-1937 populations.^7^ To do so, we aggregate by state of birth, rather than state of residence, for respondents born in the 5 years before (1932–1936) our treatment date of 1937. This results in a pre-period measure of genetic susceptibility to bacterial infections for the 48 contiguous US states.

While imperfect, self-reported ancestry represents our only option to measure ethnic compositions (Kaseniit et al., 2020; Ramos et al., 2016). Errors associated with self-reported ancestry should not bias our estimations, however. This error in reporting is likely unrelated to our hypothesized relationship between HLA susceptibility and bacterial mortality rates that we explore.

Our primary measure of susceptibility within the HLA system considers reported ancestry groups to be segregated (i.e., no intermarriage between ethnicities), so that a state’s HLA susceptibility is simply a weighted average of the HLA homozygosity from the state’s ancestral composition. In other words, our primary measure considers diversity *within* ancestral populations, not a measure across ancestries. For example, if a state were entirely composed of French (the most resistant ethnic group), our hypothesis is that this state would have the lowest level of infectious disease mortality prior to antibiotics, and as a result, would have the smallest declines post-treatment. The inclusion of additional ethnic groups to this hypothetical state would *lower* our measure of HLA diversity, since the added groups have lower ancestry-specific HLA diversity and our base measure is simply the weighted average of each ethnicity’s HLA score. This implies states that are composed of ethnic populations with low levels of HLA diversity will in aggregate have a high HLA susceptibility score. Increasing the total number of ethnicities within a state *will not* alter our state-level measure of HLA susceptibility, suggesting ethnic diversity is not driving variation in HLA susceptibility. Indeed, the cross-state correlation between HLA susceptibility and ethnic diversity is *positive* and weakly significant (p = 0.059). In constructing our resistance measure within ancestral groups, we are assuming that social and disease networks are segmented; an assumption that is further discussed and tested in Section 3.2.

### 2.2. Outcome and control variables

Bacterial mortality rates (per 100 K) are from the annual Vital Statistics of the United States. The mortality rate from each specified infectious disease is by state and is composed of typhoid fever, scarlet fever, pertussis (whooping cough), tuberculosis, diphtheria, flu and pneumonia, diarrhea and enteritis, syphilis, and maternal mortality (a proxy for puerperal fever).^8^ Our primary sample period is from 1932 to 1942, which is centered around the introduction date of sulfa drugs in 1937; however, we also look at effects for an extended sample of 1900–1970.

Our base specification comprises a difference-in-differences framework that includes state and year or year-by-census division fixed effects. Controls are piecemeal introduced in three sets and attempt to account for differential post-1937 trends that may be associated with HLA susceptibility. The first set of controls are climatic: the annual average of monthly temperature and precipitation. The second set is made up of demographic controls—the fraction of a state’s population that is Black in 1936, the fraction of a state’s 1936 population that is foreign born, the urbanization rate in 1936, the state’s population in 1936, the number of state-level in and out migrants between 1935 and 1940, and a measure of ethnic fractionalization based on the census-level reported ethnicity used in the construction of our measure of HLA homozygosity (Lleras-Muney 2002, 2005; Census Board 1943; Ruggles et al. 2017; Turner et al. 2007). The third set of controls comprises infrastructure measures—schools per square mile in 1936, education expenditures per capita in 1936, hospitals per square mile in 1936, physicians per capita in 1936, and state-level real income in 1936 (Lleras-Muney 2002, 2005; Turner et al. 2007). Further definitions, sources, and summary statistics for all variables can be found in the appendix.

### 2.3. Empirical strategy

Our empirical approach is similar in spirit to the difference-indifferences estimations of Jayachandran et al. (2010) with two main differences. First, we do not stack within year bacterial infections versus non-bacterial infections and measure treatment differences. Instead, we examine the relative change across states in the decline of post-1937 bacterial mortality by HLA susceptibility; however, we do show for all specifications that this post-1937 decline by HLA heterozygosity is not seen in the mortality rate from all other causes absent bacterial infections. Second, we estimate the mean level decline tied to HLA susceptibility following the 1937 intervention.^9^ This contrasts with Jayachandran et al. who show a differential *linear trend* post 1937. We too estimate a statistically significant trend-break (see Appendix Table A4 and A5) but estimating a simple level difference pre versus post 1937 that is tied to a state’s HLA diversity is more aligned with our hypothesis.

Our base difference-in-differences specification compares the relative change in mortality in the post-innovation period (to the pre-innovation period) from a state’s measured genetic susceptibly to infectious disease. Expected homozygosity within the HLA system gives susceptibility to infectious disease and treatment; therefore, HLA susceptibility measures intensity of the 1937 treatment. States with more similarity within the HLA system will be less resistant to infectious disease in the pre-innovation period and are hypothesized to benefit more from the introduction of sulfa drugs. A common treatment date (i. e., 1937) is used in order to eliminate potential bias from endogenous uptake of sulfa drugs.

Formally, our estimation is given by:

(2) $$y_{it}=\alpha +\beta_{1}\text{ std}.HLA_{i}\times I_{t}^{\text{post}}+\beta_{4}\;{\text{Climate}}_{it}+\sum_{j}\beta_{j}X_{i}\times I_{t}^{\text{post}}+\gamma_{i}+\gamma_{t}+\varepsilon_{it}$$

We consider two outcome variables for a panel of *i* contiguous US States across *t* years (our base panel is from 1932 to 1942): the bacterial mortality rate and the all-cause mortality rate less bacterial mortality (referred to as residual mortality).^10^ For ease of interpretation, HLA susceptibility, or *HLA*_*i*_ is standardized to a mean of 0 and standard deviation of 1, and $I_{t}^{\text{post}}$ is an indicator for years post-1937. Annual average monthly temperature and precipitation are represented by *Climate*_*it*_. To account for characteristics that may be associated with HLA susceptibility and the corresponding post-1937 decline in bacterial mortality, $X_{i}\times I_{t}^{\text{post}}$ controls for a number of time invariant pre-period controls interacted with the post-1937 indicator. State and year (or year-by-census-division) fixed effects are represented by *γ*_*i*_ and *γ*_*t*_, respectively.

Our focus is on measuring the differences in the post-1937 decline in the bacterial mortality rate tied to HLA susceptibility. We hypothesize a *negative* relationship between these two variables: higher genetic susceptibility is associated with a larger decline in the mortality rate from bacterial infections. Therefore, our coefficient of interest, *β*_1_, is hypothesized to be negative. The unadjusted relationship is presented by Fig. 1a, which shows the decline in bacterial mortality for states HLA susceptibility quartiles. This figure supports our primary hypothesis by showing that less diverse, or more susceptible, states (i.e., HLA quartile 1) had a higher initial level of bacterial mortality and a larger post-1937 decline.

## 3. Results

### 3.1. Baseline results

Our baseline difference-in-differences (DD) estimations are given in Table 1. Panel A of Table 1 regresses bacterial mortality, while Panel B serves as a placebo test by showing the larger decline in bacterial mortality is not seen in the mortality rate for other causes.

Column (1) of Panel A gives bivariate DD estimates, controlling only for state and year fixed effects. The negative and statistically significant (p < 0.01) coefficient of interest suggests that states that are one standard deviation more susceptible to bacterial infection in the pre-period experienced roughly 14 fewer annual deaths (per 100 K) from bacterial infections following the 1937 intervention. This effect remains robust when including additional controls in columns (2)–(5). Column (2) includes regional fixed effects (interacted by year) and annual climatic controls. Column (3) adds state demographic controls to the estimates of column (2), and column (4) includes infrastructure controls to the specification of column (2). All controls are included in column (5) for the 1932–1942 panel with little effect on the magnitude or significance of the coefficient of HLA diversity. In short, states with a one standard deviation larger pre-treatment HLA susceptibility had 13–17 fewer annual bacterial deaths (per 100 K) for years 1937–1942 compared to the pre-period of 1932–1936 than states with average levels of susceptibility. Note that the average reduction in bacterial deaths in this period was approximately 53 per 100 K.

Column (6) extends the sample period to show that the effects are not localized to a strict time horizon around 1937. Extending the sample period to 1900–1970 leads to a similar negative coefficient for those states that were more susceptible in the pre-period. The magnitude, however, is much larger given the rollout of additional antibiotics after 1937 (notably penicillin in 1945) and the general decline in bacterial deaths across the 20th century. Specifically, those states that were one standard deviation more susceptible had roughly 66 fewer annual bacterial deaths (per 100 K) in years 1937–1970 compared to the pre-period of 1900–1936.

Panel B of Table 1 replicates the estimations of Panel A, replacing the bacterial mortality rate with the all-cause mortality rate less bacterial infections (i.e., residual mortality). As shown, HLA susceptibility has no statically significant differential association with this residual mortality rate following the 1937 intervention. The magnitude of the coefficient of HLA homozygosity in Panel B is like the estimates of Panel A, but this is due to the larger mortality rate for residual mortality. In other words, the point estimate decline in Panel A is associated with roughly a 10 % decline in the bacterial mortality rate, while for Panel B, the estimated decline from HLA susceptibility is roughly 1 % of the mean residual mortality rate.

**Fig. 2.**
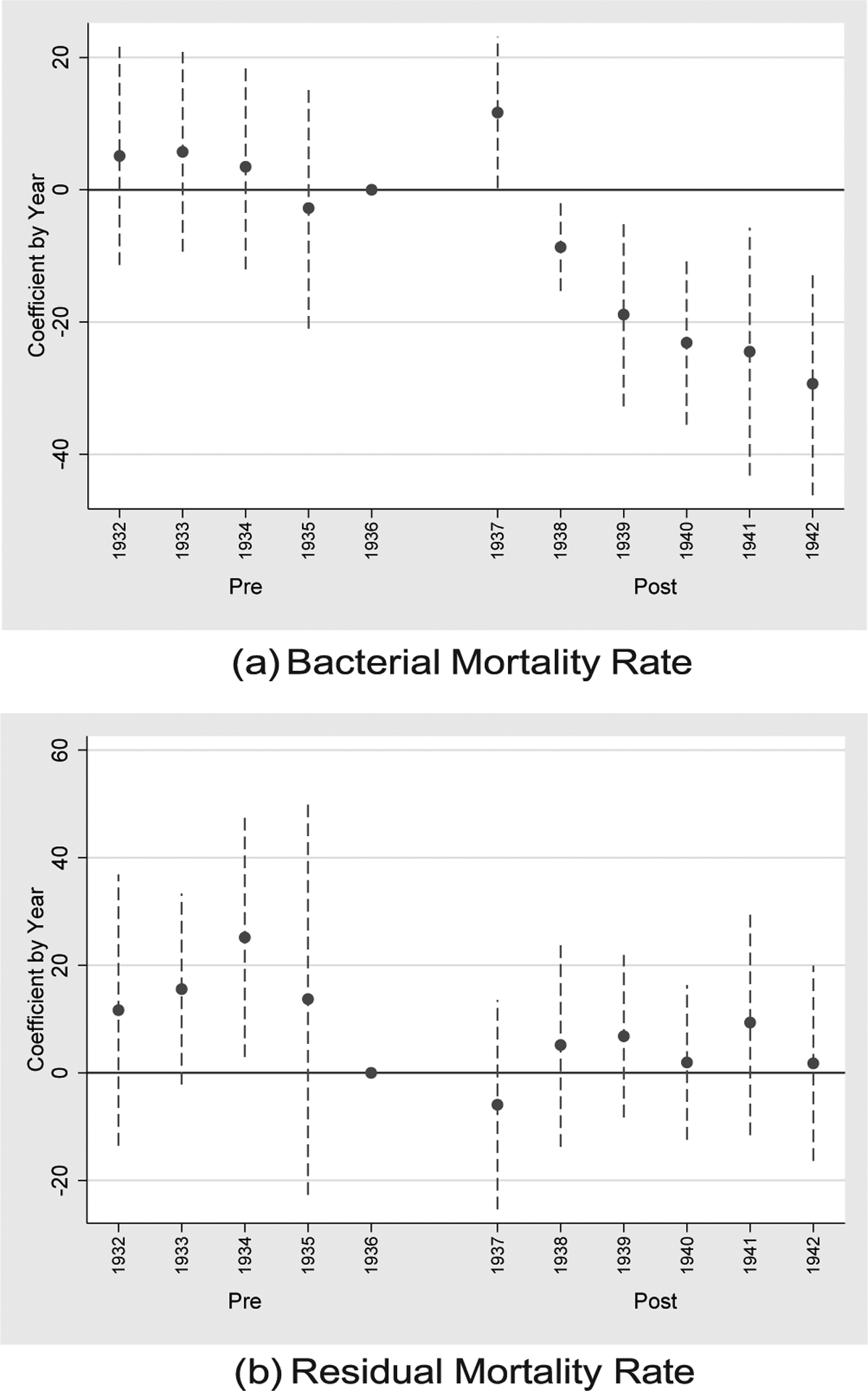
Effect of HLA Susceptibility by Year. Notes: Sub-figure (a) plots the annual coefficient (relative to 1936) of HLA susceptibility on the bacterial mortality rate. The specification follows that of column (5) of Table 1 and shows the negative post-1937 is driven by years 1938 onward. No significant effects are seen for the periods prior to 1936, suggesting no violation of the parallel pre-trends assumption. Sub-figure (b) plots the annual coefficient of HLA susceptibility, replacing bacterial mortality with the residual mortality rate; no clear pre- or post-1937 effect is seen relative to that of 1936.

The estimates of Table 1 are mirrored in Fig. 2, which plots the annual effect of HLA susceptibility on the bacterial mortality rate (subfigure a) and the residual mortality rate (subfigure b). The estimates annualize our coefficient of interest (HLA susceptibility) by interacting with year indicators, omitting the year prior to treatment, 1936. Otherwise the empirical specification is identical to that of column (5) of Table 1. As seen in subfigure (a), HLA susceptibility has no statistically significant differences in its relationship to bacterial mortality during the pre-period relative to 1936, suggesting pre-trends are not biasing our estimates. Following 1937, however, the negative association between HLA susceptibility appears. For residual mortality in subfigure (b), HLA susceptibility is insignificantly different than the 1936 association for all years except 1934. We consider the 1934 significant difference to be attributable to noise: year-by-year estimates show that HLA homozygosity is insignificantly associated with residual mortality in both 1934 and 1936, but the point estimate is relatively large and noisy for 1934.

### 3.2. Mixed HLA homozygosity

Our baseline approach considers ethnic populations in the 1930s US to be fully segregated. This is obviously incorrect, but we assume that this is closer to the truth at the time. An alternative approach is to construct HLA homozygosity from *state-level gene frequencies*, instead of ancestral group gene frequencies. Measuring homozygosity in this way assumes a common, fully integrated state-level population, instead of a state being made up of several distinct ancestral groups. We consider our base segregated measure and the mixed measure of HLA diversity to be two extremes on the spectrum of ethnic interactions.^11^

As a simple example of the construction of a mixed population score, assume a state is made up equally of two ethnicities: Ethnicity A and Ethnicity B. Also assume that at a locus all members of Ethnicity A have a “T” variant, while all members of Ethnicity B have an “C” variant. If we consider these populations to be segregated and since each has no variation within the example locus, expected homozygosity would be 1 (or heterozygosity = 0). If instead we consider Ethnicity A and B to be fully integrated, then 50 % of the state population would have variant “T” and 50 % would have the “C” variant, leading to an expected homozygosity of 0.5.

In short when considering mixed ethnicities, states with more ethnic diversity tend to have more diversity within the HLA system. For example, Louisiana has the highest amount of diversity for the mixed ethnic score but is 32nd for the segregated score. Indeed, the highest 8 scores for the mixed HLA diversity score belong to states in the South. Therefore, when considering the mixed score, it is imperative to control for regional differences in the US that are associated with ethnic diversity and differences in mortality rates.

**Fig. 3.**
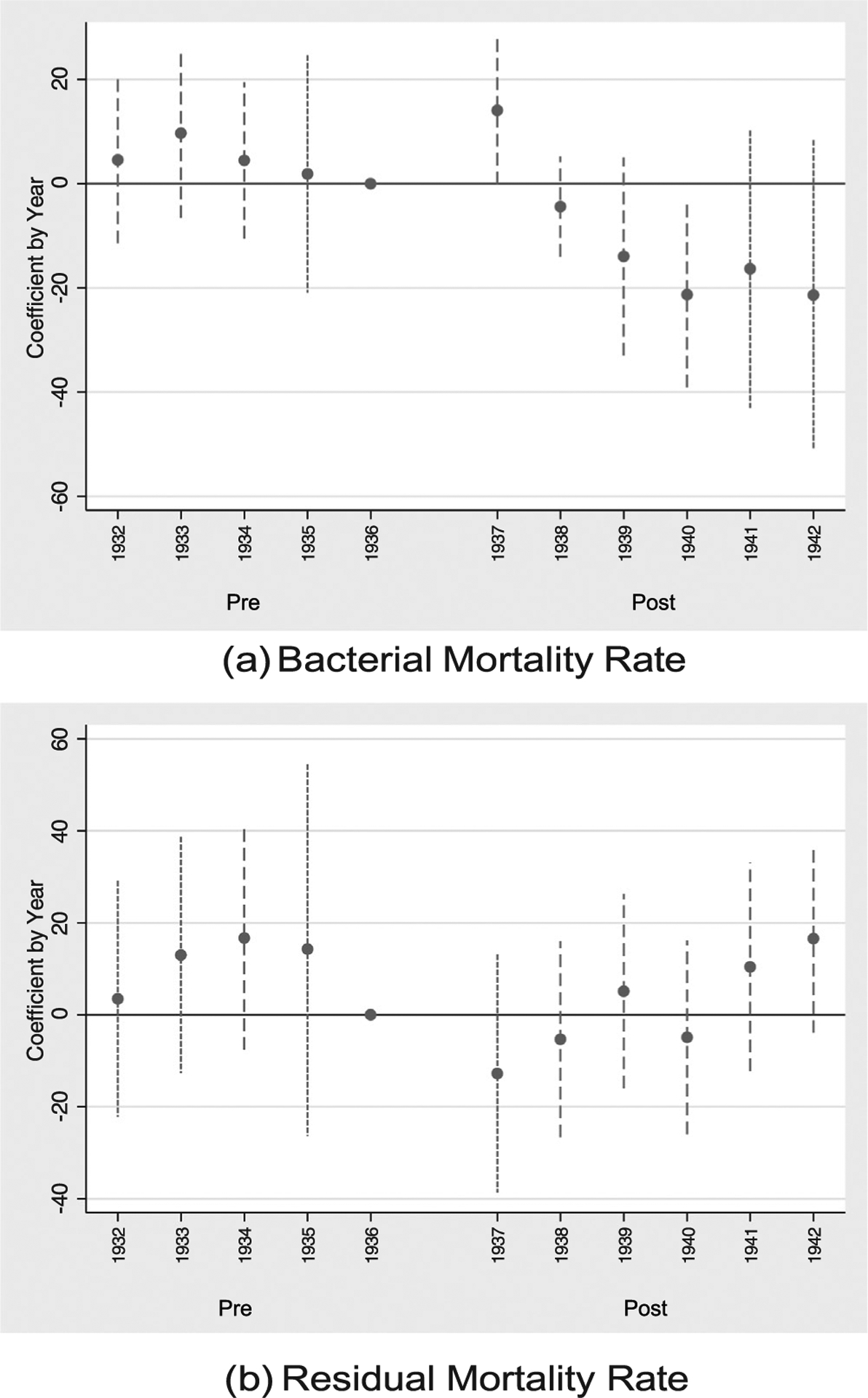
Effect of HLA Susceptibility by Year. Notes: This figure replicates Fig. 2, replacing the segregated measure of HLA susceptibility with a measure that considers ancestral/ethnic groups to be fully mixed. Sub-figure (a) plots the annual coefficient (relative to 1936) of mixed HLA susceptibility on the bacterial mortality rate. The specification follows that of column (5) of Table 2 and shows the negative post-1937 is driven by years 1938 onward. No significant effects are seen for the periods prior to 1936, suggesting no violation of the parallel pre-trends assumption. Sub-figure (b) plots the annual coefficient of mixed HLA susceptibility, replacing bacterial mortality with the residual mortality rate; as with the segregated measure, no clear pre- or post-1937 effect is seen relative to that of 1936.

Table 2 and Fig. 3 replace our primary measure of HLA susceptibility, which assumes segregated ethnic populations, with the mixed measure of diversity discussed above. The table and figure are otherwise identical. Panel A of Table 2 regresses the bacterial mortality rate, and Panel B regresses the residual mortality rate, and corresponding event figure plots are respectively given by sub-figures (a) and (b) of Fig. 3.

The estimated coefficients of the mixed HLA susceptibility measure are very similar to the segregated measures of Table 1, except for column (1) which doesn’t account for regional differences across the US. For the base specification given by column (5), a one standard deviation increase in mixed HLA susceptibility is associated with roughly 15 fewer deaths per year (per 100 K) from bacterial infections, an estimate similar to the 17 fewer deaths for a similar change in segregated HLA susceptibility. As in Table 1, the annual decline becomes relatively larger when considering the longer panel from 1900 to 1970: a one standard deviation increase in pre-period mixed HLA susceptibility is associated with 64 fewer deaths (per 100 K) per year from bacterial causes. Finally, the estimates from Panel B again suggest that the role of HLA susceptibility is tied only to declines in deaths from bacterial causes and are not tied simply to differential trends in mortality rates following the 1937 intervention.

### 3.3. Splitting the sample by european ancestry

As shown in Appendix Table A2 and as argued in Cook (2015), European ancestry has a strong correlation with HLA susceptibility, leading to a concern that our analysis is simply capturing broad racial differences. In part this is addressed by our set of controls, which include the fraction of the state’s 1936 population that is Black. To more fully establish that HLA susceptibility is not simply accounting for broad racial differences, we split the sample of states by the fraction that are of European ancestry, or white. If we are simply picking up a differential response in bacterial mortality from majority white vs. non-white states, then this split should lead to a smaller coefficient. This is tested in Table 3. Columns (1)–(4) examine the prior base specification (col. (5) and (6) of Tables 1 and 2) association between segregated and mixed HLA susceptibility for the 1932–1942 panel and the 1900–1970 panel.

As seen in Table 3, the negative coefficients estimated in Tables 1 and 2 are mostly driven by states with smaller white majorities. From column (1), in those states with less than median European ancestry, a one standard deviation in HLA susceptibility is associated with a reduction of roughly 36 deaths from bacterial infections (per 100 K) a year; the magnitude of this effect is roughly twice that as is estimated in Table 1. Column (5) shows the effect for states with larger European fractions is roughly equal to the estimates of Table 1, but statistical significance is lost due to noisier estimation in the smaller sample and in the reduced amount of variation in the HLA measure.^12^ A similar effect is seen for the longer panel estimates of columns (2) and (6) and for the mixed HLA susceptibility score in columns (3)–(4) and (7)–(8).

In summary, our estimated decline in the bacterial mortality rate is primarily driven by states with larger minority populations, and not states with higher ancestral European populations that also contain more resistance from the HLA system. It is clear, however, that our estimated effect is not coming from differences between the majority white states vs. states with greater minority populations. Instead, it is differences in the composition associated with states that have a lower concentration of the white population that are driving our estimated effects.

## 4. Socioeconomic effects

It is worth exploring whether the differential decline in bacterial mortality by state HLA susceptibility is also associated with differential socioeconomic effects, particularly those tied to the labor force. In doing so, we formulate two hypotheses that separately consider immediate, contemporary effects to workers and the cumulative effects of lessened infant mortality, greater parental investment, and less morbidity during formative childhood years.^13^

Our first hypothesis is that the introduction of antibiotics (i.e., sulfa drugs) would reduce mortality that is more prevalent in older workers, effectively aging the contemporary workforce, and this ageing of the workforce is more pronounced in states that had larger declines in bacterial mortality or in states that were more susceptible to bacterial infections prior to treatment. In other words, we expect a relative and immediate increase following treatment in the average age of the workforce that is tied to a state’s HLA susceptibility.

The second hypothesis considers cumulative life-long effects of being born into an environment that is less impacted by infectious disease. The idea being that the parents of those born after treatment face Beckerian-type incentives, where the reduction in infectious disease reduces the risks—i.e., premature death, high levels of morbidity, etc.—associated with parental investments and increases investment in children. In turn, these greater investments manifest later in life through the acquisition of schooling. Both hypotheses are explored further and tested below, where it is shown that there are indeed relative increases in the age of the work force and a greater accumulation of schooling in more HLA susceptible states after 1937.^14^

### 4.1. Contemporary effect: worker age

The mortality curve of the infectious disease in question primarily affected the young and old (Armstrong et al., 1999). We first examine immediate effects from a reduced burden in infectious disease following the introduction of effective sulfa drugs in 1937. In doing so, we are effectively testing the effect of those already in the labor force. Our estimation strategy is consistent. We employ the same state-by-year panel as used in Tables 1 and 2 but replace bacterial/residual mortality with the state-year average age of those 16–65 (Turner et al., 2007).^15^

These results are presented in Table 4 for both the measure of HLA susceptibility calculated assuming segregated ancestral/ethnic groups (Panel A) and the similar measure calculated assuming mixed ancestral/ethnic groups (Panel B). Columns are consistent with prior estimation with the piecemeal introduction of previously defined sets of control variables.

**Fig. 4.**
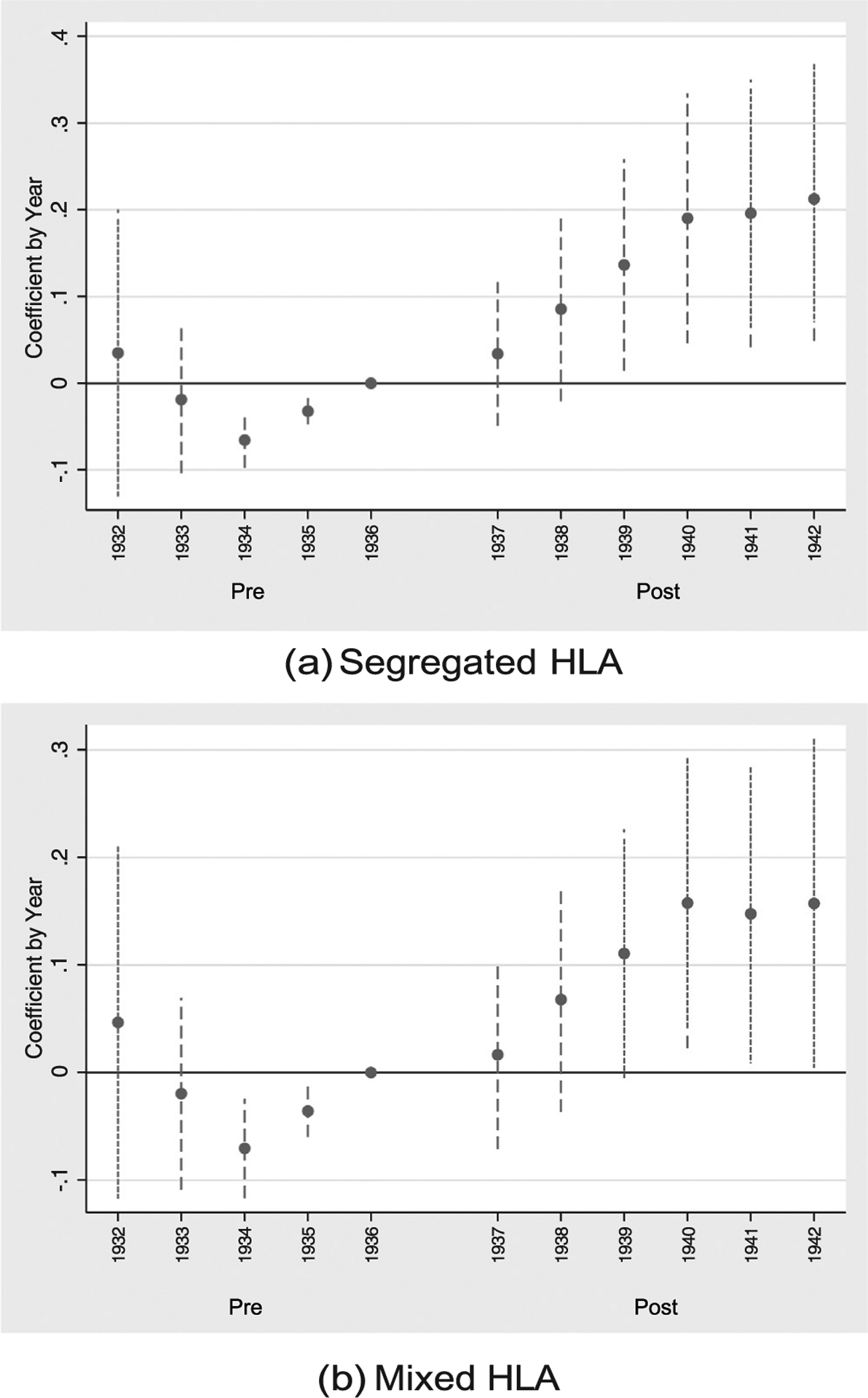
Effect of HLA Susceptibility on Age of Labor Force by Year. Notes: This figure plots annual coefficients relative to 1936 for the empirical specification of column (5) of Table 4. Sub-figure (a) plots the annual coefficient for the segregated measure of HLA susceptibility and shows positive effects on age following the introduction of sulfa drugs in 1937. No clear positive pre-trend is seen prior to 1936. Sub-figure (b) plots the annual coefficient for the mixed HLA susceptibility measure, showing very similar coefficients as those of the segregated measure.

For the segregated measure of HLA susceptibility in Panel A, states that are one standard deviation more susceptible to bacterial infections are shown to have an increased average age of 0.16–0.18 years for the short panel of 1932–1942 (columns (1)–(5)). All estimated coefficients are significant at the 1 % level. Annual estimates, or an event figure, from the full specification of column (5) are given in Fig. 4a. There is a slight pre-trend in the two years prior to 1936, but clear and positive relative effects of HLA susceptibility only after treatment in 1937.

The estimated effect for the longer 1900–1970 panel, which is given by column (6) of Table 4, is similar in magnitude to that of the shorter panel but less precisely estimated. Interestingly, the estimated coefficient does not get larger as with bacterial mortality, suggesting the absence of accumulation. In other words, the relative age adjustment is mostly centered around the introduction of treatment in 1937 and not becoming more pronounced over time; however, due to the imprecision of the estimated coefficient, we cannot rule out that there is indeed no cumulative benefit as seen with bacterial mortality.

The estimates for the mixed measure in Panel B of Table 4 and Fig. 4b are very similar to those of the segregated measure. The exceptions being a slightly smaller magnitude when including all controls, and no significant effect when omitting census division by year effects. The event figure given by Fig. 4b also looks identical to that of the segregated measure in Fig. 4a, showing a slight pre-trend in the 2 years preceding treatment and positive relative effects post treatment.

In addition to average age, we have also looked at worker experience. Given the time frame, we consider age and experience to be very similar measure. Indeed, for 1932–1942 the correlation is 0.88, and estimates are very similar in magnitude, direction, and the time-of-effect (as seen in an event figure) when considering experience as the dependent variable.

The estimates of Table 4 and Fig. 4 show that workers in states that were more susceptible to infectious disease through ancestrally determined genetic resistance had relatively larger immediate increases in the age and experience of their workforce. We next examine whether the larger relative improvement in the disease environments of these states lead to comparative, relative increases in human capital, measured by years of schooling.

### 4.2. Cohort effect: more education

To examine potential cumulative effects to education, we take a slightly different approach to our state-by-year panel. The primary change is to explore differences across *birth cohorts* in place of contemporary annual differences. To do so, we once again use the 5 % Census Samples for 1980, 1990, and 2000 to create an individual (and aggregated state) level panel based on birth year and birth state. We then again estimate differential responses to the post-1937 treatment by state-level HLA susceptibility. Our primary estimation strategy examines differences in *individuals* across states to more fully control for individual characteristics that are associated with differences in schooling—i.e., sex, urban/rural status, race, and age; however, aggregating years of schooling by birth year and birth cohort produces similar findings.

Thus, our use of birth cohort differences effectively examines in utero differences in exposure to a lessened disease environment, with the degree of this lessening tied to HLA susceptibility. In other words, there are relatively larger benefits from treatment for those in utero in a more HLA susceptible states. This test is akin to that of Almond (2006), who documents clear association between more in utero exposure to disease and lessened educational attainment later in life. Our approach of examining in utero differences is also narrow in that a lessened exposure to infectious disease early in life (but after in utero) also likely had beneficial effects to educational attainment. This narrow focus is further addressed and we believe, present when looking at event figures when examining the relative effect of HLA susceptibility on later life schooling compared to the year prior to treatment.

Unobserved cohort selection (or culling) is also likely; however, we believe this selection would lead to a negative bias, attenuating our coefficient of interest. Prior to treatment, there is a negative state-level correlation between income and bacterial mortality (−0.24 for years 1932–1936). It’s safe to assume there is likely a similar correlation between individual income and bacterial mortality. Therefore, there were likely a greater number of deaths among individuals (or their parents) with a lower socioeconomic status prior to treatment, removing them from our sample and therefore increasing pre-treatment levels of education. Following treatment, we can assume that these poorer individuals that would have died (and been removed from our sample) now live and accumulate fewer years of schooling than better-off peers. This, in effect, implies lessened relative growth, or even declines, in measured schooling for states with high pre-period mortality from bacterial infections, the opposite of our findings.

The estimates from the individual panel are given in Table 5. Although our panel is now at the individual level, state-level controls are piecemeal introduced identically to that of Tables 1 and 2. One differences is that we now include a set of individual controls along with the set of state-level demographic controls. These individual controls include indicators for sex, urban/rural status, race, and age. Again, Panel A considers the segregated measure of HLA susceptibility, while Panel B considers the mixed measure.

For the segregated measure, individuals born in states one standard deviation more susceptible to infectious disease are shown to have a 0–07 year (Table 5, column (5), Panel A) gain following the introduction of sulfa drugs in schooling compared to those at mean susceptibility. This corresponds to roughly 16 % of the average pre vs. post gain in schooling in the 1932–1942 birth cohort panel. For the longer panel of column (6), individuals in states one standard deviation above mean are shown to accumulate 0.2 more years following treatment; this corresponds to roughly 11 % of the average pre vs. post change in years of schooling. When including necessary controls, estimates for the mixed measure in Panel B are very similar magnitude and significance.

**Fig. 5.**
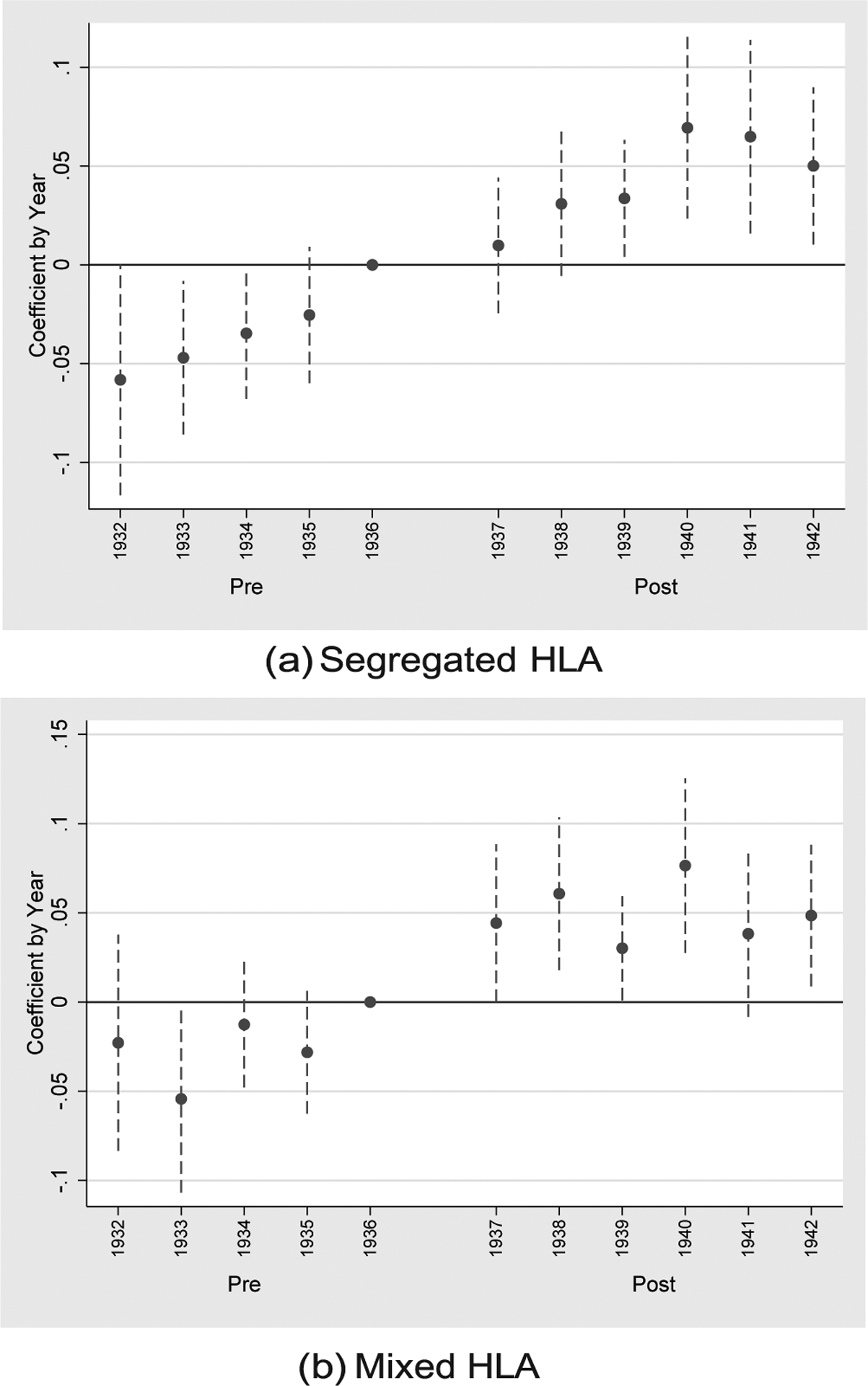
Effect of HLA Susceptibility on Education by Birth Year, 1932–1942. Notes: This figure plots annual coefficients relative to 1936 for the empirical specification of column (5) of Table 5. Sub-figure (a) plots the annual coefficient for the segregated measure of HLA susceptibility and shows positive effects on accumulated years of schooling following the introduction of sulfa drugs in 1937. A positive pre-trend is seen prior to 1936; this is further explored in Fig. 6. Sub-figure (b) plots the annual coefficient for the mixed HLA susceptibility measure, showing very similar coefficients as those of the segregated measure following treatment but shows no pre-trend differences before treatment.

Event figures or annual estimates (relative to the year prior to treatment) for the specification of column (5) of Table 5 are plotted in Fig. 5. Fig. 5a plots estimates for the segregated measure, and Fig. 5b plots estimates for the mixed measure. Fig. 5a shows a clear pre-trend; more susceptible birth cohorts are shown to have increases in schooling prior to treatment in 1937. This pre-trend, however, may be due (at least in part) to beneficial effects of treatment on those early in childhood. In other words, the cumulative benefits of a life-long exposure to an environment that is less burdened by infectious disease may be present in those early in life (for example, those under 5 years old). This benefit for those born prior to treatment but are still in-part treated would appear as a pre-trend.

**Fig. 6.**
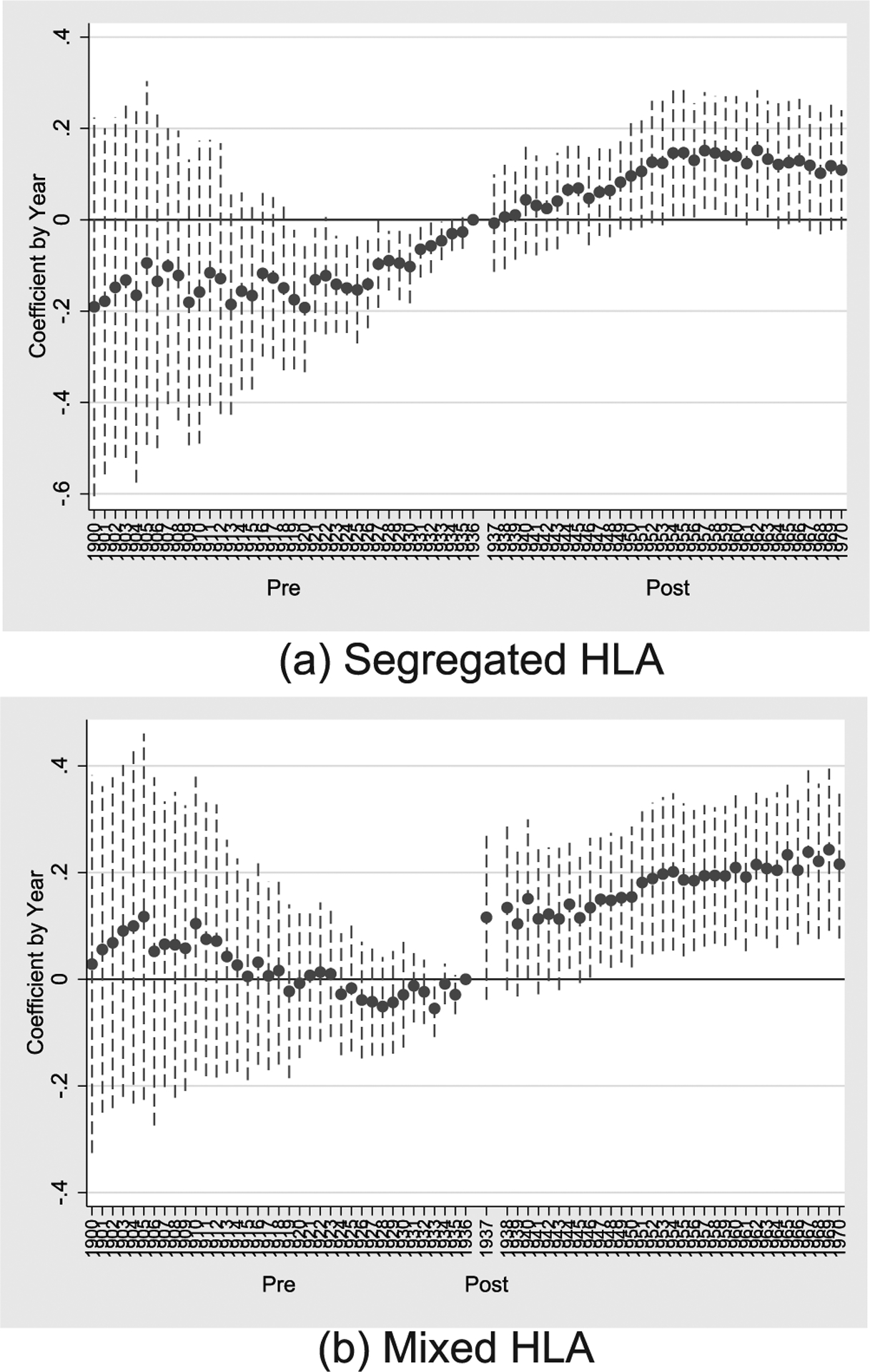
Effect of HLA Susceptibility on Education by Birth Year. Notes: This figure again plots annual coefficients relative to 1936 for the empirical specification of column (5) of Table 5. The sample is extended to 1900–1970, however, to show the pre-trends observed in Fig. 5 are likely due to treatment effects spilling over into those born before 1937 but still in early childhood. Sub-figure (a) plots the annual coefficient for the segregated measure of HLA susceptibility. Sub-figure (b) plots the annual coefficient for the mixed HLA susceptibility measure and shows no. pre-trends.

To further explore this, we plot annual estimates for the longer panel of column (6) of Table 6 in Fig. 6. Fig. 6a, which (6). Which considers the segregated measure of HLA susceptibility, shows that the effect of HLA susceptibility is virtually unchanged for birth cohorts born between 1900 and 1931, but beginning in 1932 (when individuals are 5 years old at the time of treatment), there are increasing positive effects from living in more susceptible states. This plot gives credence to the idea of treatment effects spilling over to young children born prior to treatment. Concerns of pre-trends are not present when considering the mixed measure of HLA susceptibility.

## 5. Conclusion

This paper explores the possibility that population genetic phenomena may interact with medical innovation to produce heterogenous treatment effects. Indirect evidence of this possibility was first documented in Cook (2015) but did not focus on a specific medical innovation. In this paper, we present evidence that the invention of sulfa drugs to combat bacterial infections in the early 20th century produced differential effects on bacterial mortality that varied by area-level genetic endowments. The logic of the result is that prior to the onset of medicine, inherent (genetic) resistance mechanisms played an important role in determining the virulence of infectious disease. Historical exposure, while harmful at the time, imprinted a legacy of resistance (or susceptibility) on ancestral groups or ethnicities. Thus, we use these imprinted differences in the HLA system to document that states composed of ancestral populations that were more susceptible to bacterial infections had greater declines in bacterial-caused deaths following the introduction of sulfa drugs in 1937.

Our estimations and figures show clear pre-1937 differences in bacterial mortality tied to HLA susceptibility that begin to dissipate following the rollout of sulfa drugs. Specifically, susceptible states experienced 13–17 fewer deaths from bacterial mortality (per year and per 100 K) following the 1937 intervention compared to those of mean susceptibility. Importantly, this decline is limited only to infectious disease mortality; no differential decline is observed when examining all other causes of death.

Given this differential decline, we also explore contemporary and cumulative effects to the labor market. The mortality curve associated with the bacterial infections in question show the greatest benefits to life would be among the old and young. Contemporary estimates (i.e., the effect for older workers) show a relative aging in the workforce for states more exposed to infectious disease prior to treatment, and cumulative benefits (i.e., the effect on the young) show a greater acquisition of schooling to individuals in more susceptible states. This suggests effects to output may be tied to the longer-term acquisition of human capital, which may be understated in other studies looking at immediate benefits from the eradication of infectious disease (e.g., Acemoglu and Johnson 2007; Hansen 2014). More work is needed to examine this potential channel on output.

We recognize that our measure of inherent resistance has limitations. Measurement errors are likely due to our primary measure being based on self-reported ancestry. We have no reason, however, to assume that this is anything other than classical measurement error. Additionally, we focus strictly on mortality and are unable to account for the harmful effects of morbidity.^16^ This focus on mortality is also likely centered on the young and old, preventing us from extending our results to more general health improvements that have a more demographically uniform distribution of health gains or losses. With those limitations recognized, our results support the claim that some modern medicines have worked as a substitute for ‘natural resistance’ to infectious disease, with the implication that the overall value of these innovations are heterogenous and may not be accurately measured by previous explorations of average impacts on population health and may also require caution in extrapolating impacts between populations.

## Supplementary Material

Appendix A - 2

Appendix A - 1

## Figures and Tables

**Table 1:** Baseline panel estimates: segregated HLA similarity.

	Dependent variable: by panel					
	(1)	(2)	(3)	(4)	(5)	(6)
Panel A. Bacterial Mortality Rate						
Post-1937 × Std. HLA	−14.2526***	−12.8817***	−16.8477***	−13.7968***	−17.4756***	−66.1424***
	(3.2917)	(2.7199)	(2.4019)	(2.4426)	(1.8746)	(4.7794)
Observations	527	527	527	527	527	2711
Years	1932–1942	1932–1942	1932–1942	1932–1942	1932–1942	1900–1970
Mean Bacterial Mortality Rate	191.51	191.51	191.51	191.51	191.51	180.57
Pre-period (Year<1937)	220.47	220.47	220.47	220.47	220.47	331.02
Post-period (Year≥1937)	167.48	167.48	167.48	167.48	167.48	81.10
Panel B. Residual Mortality Rate						
Post-1937 × Std. HLA	−9.7550	−15.1863	−18.1057	−11.7161	−10.5367	−17.3851
	(8.7384)	(10.3424)	(10.8717)	(10.8586)	(9.8184)	(20.7913)
Observations	527	527	527	527	527	2711
Years	1932–1942	1932–1942	1932–1942	1932–1942	1932–1942	1900–1970
Mean Residual Mortality Rate	890.88	890.88	890.88	890.88	890.88	895.27
Pre-period (Year<1937)	888.82	888.82	888.82	888.82	888.82	906.77
Post-period (Year≥1937	892.58	892.58	892.58	892.58	892.58	887.66
Controls:						
Fixed Effects:						
State	Y	Y	Y	Y	Y	Y
Year						
Year × Census Division	Y	Y	Y	Y	Y	Y
Time-varying:						
Mean Temperature		Y	Y	Y	Y	Y
Mean Precipitation		Y	Y	Y	Y	Y
Time-invariant × Post-1937						
Demographic Set			Y		Y	Y
Infrastructure Set				Y	Y	Y

Summary & Notes: This table represents our baseline estimations by showing states that are more susceptible to infectious disease through less genetic diversity (or more similarity) within the HLA system have a larger decline in bacterial morality rates following the intervention of sulfa drugs in 1937. HLA susceptibility, or homozygosity, is calculated within ethnic groups and averaged to the state level, and then standardized to a mean of 0 and standard deviation of 1. Panel A regresses the bacterial mortality rate, and Panel B regress the overall mortality rate less bacterial rate (i.e., the residual mortality rate). The demographic set of controls include the fraction of a state’s population that is Black in 1936, the fraction of a state’s 1936 population that is foreign born, the urbanization rate in 1936, the state’s population in 1936, the number of state-level in and out migrants between 1935 and 1940, and a measure of ethnic fractionalization based on the census-level reported ethnicity. The set of infrastructure controls includes education expenditures per capita in 1936, schools per square mile in 1936, hospitals per square mile in 1936, physicians per capita in 1936, and state-level real income in 1936. Standard errors are clustered by state. Statistical significance is denoted by *, **, and ***, representing significance at the 10 %, 5 %, and 1 % levels, respectively.

**Table 2:** Baseline panel estimates: mixed HLA similarity.

	Dependent variable: by panel					
	(1)	(2)	(3)	(4)	(5)	(6)
Panel A. Bacterial Mortality Rate						
Post-1937 × Std. Mixed HLA	−7.6422	−12.0831***	−14.4589***	−14.0303***	−14.7498***	−63.6012***
	(5.2000)	(4.3444)	(4.9050)	(3.1647)	(3.6097)	(11.0151)
Observations	527	527	527	527	527	2711
Years	1932–1942	1932–1942	1932–1942	1932–1942	1932–1942	1900–1970
Mean Bacterial Mortality Rate	191.51	191.51	191.51	191.51	191.51	180.57
Pre-period (Year<1937)	220.47	220.47	220.47	220.47	220.47	331.02
Post-period (Year≥1937)	167.48	167.48	167.48	167.48	167.48	81.10
Panel B. Residual Mortality Rate						
Post-1937 × Std. Mixed HLA	4.5815	−2.1509	−14.7724	−3.1984	−7.9545	−7.9316
	(8.6278)	(10.3333)	(11.5536)	(10.1509)	(9.6267)	(20.8421)
Observations	527	527	527	527	527	2711
Years	1932–1942	1932–1942	1932–1942	1932–1942	1932–1942	1900–1970
Mean Residual Mortality Rate	890.88	890.88	890.88	890.88	890.88	895.27
Pre-period (Year<1937)	888.82	888.82	888.82	888.82	888.82	906.77
Post-period (Year≥1937)	892.58	892.58	892.58	892.58	892.58	887.66
Controls:						
Fixed Effects:						
State	Y	Y	Y	Y	Y	Y
Year						
Year × Census Division	Y	Y	Y	Y	Y	Y
Time-varying:						
Mean Temperature		Y	Y	Y	Y	Y
Mean Precipitation		Y	Y	Y	Y	Y
Time-invariant × Post-1937						
Demographic Set			Y		Y	Y
Infrastructure Set				Y	Y	Y

Summary & Notes: This table replicates Table 1, replacing HLA susceptibility constructed from segregated ethnic populations with a similar measure that considers fully mixed ethnic populations within a state. This mixed HLA susceptibility standardized to a mean of 0 and standard deviation of 1. Panel A regresses the bacterial mortality rate, and Panel B regress the overall mortality rate less bacterial rate (i.e., the residual mortality rate). The demographic set of controls include the fraction of a state’s population that is Black in 1936, the fraction of a state’s 1936 population that is foreign born, the urbanization rate in 1936, the state’s population in 1936, the number of state-level in and out migrants between 1935 and 1940, and a measure of ethnic fractionalization based on the census-level reported ethnicity. The set of infrastructure controls includes education expenditures per capita in 1936, schools per square mile in 1936, hospitals per square mile in 1936, physicians per capita in 1936, and state-level real income in 1936. Standard errors are clustered by state. Statistical significance is denoted by *, **, and ***, representing significance at the 10 %, 5 %, and 1 % levels, respectively.

**Table 3:** Splitting sample by European ancestry.

	Dependent variable: Bacterial Mortality Rate							
	Below Median European Ancestry				Above Median European Ancestry			
	(1)	(2)	(3)	(4)	(5)	(6)	(7)	(8)
Post-1937 × Std. HLA	−36.1483***	−93.4730***			−16.7304	−63.2002*		
	(3.0720)	(8.0868)			(15.7440)	(31.6811)		
Post-1937 × Std. Mixed HLA			−36.9196***	−91.1953***			−16.9755**	−33.1231*
			(2.7357)	(8.2171)			(7.9893)	(18.2857)
Controls:								
Fixed Effects:								
State	Y	Y	Y	Y	Y	Y	Y	Y
Year × Census Division	Y	Y	Y	Y	Y	Y	Y	Y
Time-varying:								
MeanTemperature	Y	Y	Y	Y	Y	Y	Y	Y
Mean Precipitation	Y	Y	Y	Y	Y	Y	Y	Y
Time-invariant × Post-1937								
Demographic Set	Y	Y	Y	Y	Y	Y	Y	Y
Infrastructure Set	Y	Y	Y	Y	Y	Y	Y	Y
Observations	252	1146	252	1146	264	1468	264	1468
States	23	23	23	23	24	24	24	24
Years	1932–1942	1900–1970	1932–1942	1900–1970	1932–1942	1900–1970	1932–1942	1900–1970
Mean Bacterial Mortality Rate	236.58	171.40	236.58	171.40	149.28	182.45	149.28	182.45
Pre-period (Year<1937)	268.29	334.51	268.29	334.51	173.04	326.19	173.04	326.19
Post-period (Year≥1937)	205.48	95.47	205.48	95.47	129.48	67.61	129.48	67.61

Summary & Notes: This table bifurcates the sample by the fraction of the population that is of European ancestry, or white. This is done to show that our findings are not due to the presence or absence of an overly white population. The effect of HLA susceptibility is relatively larger in states with a smaller white fraction of the population. In part, this is likely due to increased variation in our HLA homozygosity score in less white states (s.d. of HLA homozygosity is roughly 3 times larger in states with below median white fractions compared to those above median). The demographic set of controls include the fraction of a state’s population that is Black in 1936, the fraction of a state’s 1936 population that is foreign born, the urbanization rate in 1936, the state’s population in 1936, the number of state-level in and out migrants between 1935 and 1940, and a measure of ethnic fractionalization based on the census-level reported ethnicity. The set of infrastructure controls includes education expenditures per capita in 1936, schools per square mile in 1936, hospitals per square mile in 1936, physicians per capita in 1936, and state-level real income in 1936. Standard errors are clustered by state. Statistical significance is denoted by *, **, and ***, representing significance at the 10 %, 5 %, and 1 % levels, respectively.

**Table 4:** Contemporary effect: worker age.

	Dependent variable: Average Age, 16–65					
	(1)	(2)	(3)	(4)	(5)	(6)
Panel A. Segregated HLA Susceptibility						
Post-1937 × Std. HLA	0.1803***	0.1757***	0.1701***	0.1804***	0.1585***	0.1647*
	(0.0515)	(0.0424)	(0.0555)	(0.0381)	(0.0420)	(0.0823)
Observations	528	528	528	528	528	2158
Years	1932–1942	1932–1942	1932–1942	1932–1942	1932–1942	1900–1970
Mean of Dependent Variable	34.19	34.19	34.19	34.19	34.19	34.38
Pre-period (Year<1937)	33.85	33.85	33.85	33.85	33.85	32.76
Post-period (Year≥1937)	34.47	34.47	34.47	34.47	34.47	34.91
Panel B. Mixed HLA Susceptibility						
Post-1937 × Std. HLA	0.0636	0.1654***	0.1665**	0.1416***	0.1258**	0.1234
	(0.0410)	(0.0461)	(0.0650)	(0.0394)	(0.0470)	(0.0979)
Observations	528	528	528	528	528	2158
Years	1932–1942	1932–1942	1932–1942	1932–1942	1932–1942	1900–1970
Mean of Dependent Variable	34.19	34.19	34.19	34.19	34.19	34.38
Pre-period (Year<1937)	33.85	33.85	33.85	33.85	33.85	32.76
Post-period (Year≥1937)	34.47	34.47	34.47	34.47	34.47	34.91
Controls:						
Fixed Effects:						
State	Y	Y	Y	Y	Y	Y
Year						
Year × Census Division	Y	Y	Y	Y	Y	Y
Time-varying:						
Mean Temperature		Y	Y	Y	Y	Y
Mean Precipitation		Y	Y	Y	Y	Y
Time-invariant × Post-1937						
Demographic Set			Y		Y	Y
Infrastructure Set				Y	Y	Y

This table examines how the average age of the working-age population responded deferentially by HLA susceptibility to the introduction of sulfa drugs in 1937. In summary, states that were more susceptible to infectious disease prior to treatment, experienced relative gains to age following treatment. Panel A uses a measure of HLA susceptibility that assumes fully segregated ancestral populations, and Panel B uses a measure of HLA susceptibility that assumes fully mixed ancestral populations. The demographic set of controls include the fraction of a state’s population that is Black in 1936, the fraction of a state’s 1936 population that is foreign born, the urbanization rate in 1936, the state’s population in 1936, the number of state-level in and out migrants between 1935 and 1940, and a measure of ethnic fractionalization based on the census-level reported ethnicity. The set of infrastructure controls includes education expenditures per capita in 1936, schools per square mile in 1936, hospitals per square mile in 1936, physicians per capita in 1936, and state-level real income in 1936. Standard errors are clustered by state. Statistical significance is denoted by *, **, and ***, representing significance at the 10 %, 5 %, and 1 % levels, respectively.

**Table 5:** Birth cohort effect: education.

	Dependent variable: Years of Schooling (age>30)					
	(1)	(2)	(3)	(4)	(5)	(6)
	Panel A. Segregated HLA Susceptibility					
Post-1937 × Std. HLA	0.1131***	0.0633**	0.0755***	0.0619***	0.0742***	0.2018***
	(0.0151)	(0.0238)	(0.0219)	(0.0220)	(0.0187)	(0.0635)
Observations	3,383,021	3,383,021	3,383,021	3,383,021	3,383,021	18,219,922
Birth Years	1932–1942	1932–1942	1932–1942	1932–1942	1932–1942	1900–1970
Mean of Dependent Variable	13.35	13.35	13.35	13.35	13.35	13.31
Pre-period (Year<1937)	13.09	13.09	13.09	13.09	13.09	12.18
Post-period (Year≥1937)	13.55	13.55	13.55	13.55	13.55	14.10
	Panel B. Mixed HLA Susceptibility					
Post-1937 × Std. HLA	−0.0584**	0.0427	0.0734***	0.0564*	0.0731***	0.1694***
	(0.0265)	(0.0285)	(0.0198)	(0.0284)	(0.0175)	(0.0605)
Observations	3,383,021	3,383,021	3,383,021	3,383,021	3,383,021	18,219,922
Birth Years	1932–1942	1932–1942	1932–1942	1932–1942	1932–1942	1900–1970
Mean of Dependent Variable	13.35	13.35	13.35	13.35	13.35	13.31
Pre-period (Year<1937)	13.09	13.09	13.09	13.09	13.09	12.18
Post-period (Year≥1937)	13.55	13.55	13.55	13.55	13.55	14.10
Controls:						
Fixed Effects:						
State	Y	Y	Y	Y	Y	Y
Year						
Year × Census Division	Y	Y	Y	Y	Y	Y
Time-varying:						
Mean Temperature		Y	Y	Y	Y	Y
Mean Precipitation		Y	Y	Y	Y	Y
Time-invariant × Post-1937						
Demographic Set			Y		Y	Y
Infrastructure Set				Y	Y	Y
Individual Set			Y		Y	Y

Summary & Notes: Instead of contemporary relationships, this table examines life long impacts arising from the 1937 treatment and its differential impact across states. To do so, we examine how individual years of schooling (from the same 1980, 1990, and 2000 5 % census samples used to calculate the HLA score) changes by birth cohort exposure to treatment. This table shows that states that were more exposed to infectious disease by their ancestral susceptibility also experienced relative gains in their population’s years of schooling (aggregating individuals by state produces similar results). Panel A uses a measure of HLA susceptibility that assumes fully segregated ancestral populations, and Panel B uses a measure of HLA susceptibility that assumes fully mixed ancestral populations. The demographic set of controls include the fraction of a state’s population that is Black in 1936, the fraction of a state’s 1936 population that is foreign born, the urbanization rate in 1936, the state’s population in 1936, the number of state-level in and out migrants between 1935 and 1940, and a measure of ethnic fractionalization based on the census-level reported ethnicity. The set of infrastructure controls includes education expenditures per capita in 1936, schools per square mile in 1936, hospitals per square mile in 1936, physicians per capita in 1936, and state-level real income in 1936. Individual controls include indicators for sex, urban/rural status, age, and race. Standard errors are clustered by state. Statistical significance is denoted by *, **, and ***, representing significance at the 10 %, 5 %, and 1 % levels, respectively.

## Footnotes

1 Considering contemporary developing countries, which constitute a general proxy for early 20th century America, the overwhelming majority of deaths within the first year of life are due to infectious disease—primarily, diarrhea, pneumonia, and sepsis (WHO, 2010).

2 This possibility is linked to the large and growing literature on precision medicine, which seeks to match therapeutic drugs to individual patients based on patient characteristics (typically genetics) because of known heterogeneous effects of the drugs. In our case, we extend this micro idea to the macro context and consider population genetics as a source of heterogeneity for drugs that combat infectious disease.

3 This is only one possible mechanism for the selection of diversity within the HLA system. For a summary of others, please see Spurgin and Richardson (2010).

4 It is possible that other, unknown causes have shaped genetic variation within the HLA system, including both natural selection and potential sexual selection. To this point, Western European populations have more diversity within the HLA system than Middle Eastern and Southern Asian populations, despite being further along migration rates outside of Africa and a later adoption of agriculture. Unobserved differences in historic exposure to infectious disease may be playing a role. Furthermore, Out-of-Africa migration and agricultural endowments, particularly domesticated animals, may be jointly determined (Riahi, 2017, 2021).

5 A negative linear relationship exists between a population’s level of genetic diversity and the distance the population is along historic migration routes from East Africa. This relationship is due to a serial founder effect, in which emigrating (or founding) populations contain a subset of the genome of the origin population. As a result, subsequent emigration reduces genetic diversity along migration routes.

6 The average is taken for individuals that report two different ancestries. Reported ancestries from the census are sometimes country-specific and other times ethnic-specific. For example, Belgium vs. Flemish. Since most countries are composed of more than one ethnicity, individuals that report country heritage—e.g., Belgian—are generally assigned the country score, while those that report specific ethnicity—e.g., Flemish—are generally assigned ethnic scores. Given the arbitrary nature of this matching, we include every ancestry/ethnicity from the census and its corresponding match in an appendix.

7 An additional concern is that our approach in defining state-level HLA susceptibility by state-of-birth from more contemporary census data opens the possibility of selection; however, this potential selection is more likely to shift our estimated relationship *towards* zero, rather than leading to an estimated coefficient that is statistically significant from zero. For example, if more susceptible ancestral groups—or ancestral groups with lower diversity within the HLA system—die at greater rates from infectious disease in the intervening years between pre-1937 and 1980 (the first census year), then these groups would be underrepresented in our calculated HLA diversity score. This under-representation would then skew up our HLA diversity score, which is simply the weighted average for each ancestral group, creating a situation in which a high HLA diversity score is associated with a greater death rate from infectious disease. In other words, the potential selection out of vulnerable populations is associated with both an increase in mortality rates and an increase in a state’s HLA diversity score, the opposite of our hypothesis and main findings.

8 While influenza is a viral infection, flu and pneumonia are jointly reported for most years.

9 A 1938 revision to the classification of mortality causes could potentially affect our results; however, this classification would need to be correlated with HLA susceptibility, which is unlikely. Furthermore, our inclusion of census-division-by-year fixed effects accounts for broad regional differences that would pick up differences in mortality classifications (Linder and Grove, 1947).

10 In Section 4, we also consider the average age of the working age population as a dependent variable using the specification of Equation (2). Similarly, individual analysis to examine birth cohort differences in the acquisition of education following treatment follows a similar estimation equation as that given by Equation (2).

11 The correlation coefficient between the two measures of HLA diversity is 0.37.

12 The standard deviation of HLA homozygosity is roughly 3 times larger in the 24 states below the median fraction of European ancestry compared to the 24 states above median.

13 The longer, cohort-based focus, however, limits the strict causal interpretation of our estimates. It is possible that some other cultural characteristic that is related to HLA susceptibility is driving our observed increase in SES characteristics.

14 We also explore effects to income in Appendix Section A8. To summarize, there is a relative positive effect for more susceptible states—both contemporarily and for birth cohort exposure. Statistical significance, however, is mixed. We believe this generally aligns with our estimates for worker age and birth cohort years of schooling.

15 Average-age data are by decade prior to 1930 (Turner et al., 2007).

16 There is likely to be a high level of overlap between the eradication mortality from infectious disease and the eradication of morbidity from infectious disease. Furthermore, our mechanism of inherent resistance should serve to reduce morbidity in a method like its role in reducing mortality.
