# Supplementary material for: Heterogeneity in disease resistance and the impact of antibiotics in the US
Source: Econ Hum Biol. Author manuscript; Available in PMC 2023 Feb 28. (PMC9972546; doi:10.1016/j.ehb.2022.101155)
Supplement: Appendix A - 2 [file NIHMS1876438-supplement-Appendix_A_-_2.pdf]

Appendix: Matching IPUM's ancestry to HLA diversity from Cook (2015)

Census Ancestry	Ethnicity/Country from ALFRED and Cook (2015)	HLA heterozygosity	Country Code
Alsatian, Alsace-Lorraine	.5 French, .5 Orcadian	0.346954	FRA
Andorran	Italian	0.338867	ADO
Austrian	Austria (country)	0.344784	AUT
Tirolean	.5 French, .5 Orcadian	0.346954	DEU
Basque	Basque	0.318691	ESP
French Basque	Basque	0.318691	FRA
Belgian	Belgium (country)	0.346954	BEL
Flemish	.5 French, .5 Orcadian	0.346954	BEL
Walloon	French	0.353319	BEL
British	UK (country)	0.345724	GBR
British Isles	UK (country)	0.345724	GBR
Channel Islander	UK (country)	0.345724	GBR
Gibraltan	Italian	0.338867	ITA
Cornish	.5 French, .5 Orcadian	0.346954	GBR
Corsican	.5 Italian, .5 French	0.346093	ITA
Cypriot	Cyprus (country)	0.328028	CYP
Greek Cypriote	Cyprus (country)	0.328028	CYP
Turkish Cypriote	Cyprus (country)	0.328028	CYP
Danish	Denmark (country)	0.340367	DNK
Dutch	Netherlands (country)	0.346954	NLD
English	.5 French, .5 Orcadian	0.346954	GBR
Faeroe Islander	Orcadian	0.340588	GBR
Finnish	Estonian	0.331338	FIN
Karelian	Estonian	0.331338	FIN
French	French	0.353319	FRA
Lorrainian	French	0.353319	FRA
Breton	.5 French, .5 Orcadian	0.346954	GBR
Frisian	Orcadian	0.340588	DNK
Friulian	Italian	0.338867	ITA

German	Germany (country	0.340131	DEU
Bavarian	Orcadian	0.340588	DEU
Berliner	Orcadian	0.340588	DEU
Hamburger	Orcadian	0.340588	DEU
Hanoverian	Orcadian	0.340588	DEU
Hessian	Orcadian	0.340588	DEU
Lubecker	Orcadian	0.340588	DEU
Pomeranian	Russian	0.321653	DEU
Prussian	Orcadian	0.340588	DEU
Saxon	.5 French, .5 Orcadian	0.346954	DEU
Sudetenlander	Orcadian	0.340588	DEU
Westphalian	.5 French, .5 Orcadian	0.346954	DEU
Greek	Grece (country)	0.33026	GRC
Cretan	.5 Russian, .5 Italian	0.33026	GRC
Cycladic Islander	.5 Russian, .5 Italian	0.33026	GRC
Icelander	Iceland (country)	0.340588	ISL
Irish	Ireland (country)	0.346954	IRL
Italian	Italian	0.338867	ITA
Abruzzi	Italian	0.338867	ITA
Apulian	Italian	0.338867	ITA
Basilicata	Italian	0.338867	ITA
Calabrian	Italian	0.338867	ITA
Amalfin	Italian	0.338867	ITA
Emilia Romagna	Italian	0.338867	ITA
Rome	Italian	0.338867	ITA
Ligurian	Italian	0.338867	ITA
Lombardian	Italian	0.338867	ITA
Marches	French	0.353319	ITA
Molise	Italian	0.338867	ITA
Piedmontese	Italian	0.338867	ITA
Puglia	Italian	0.338867	ITA
Sardinian	Sardinian	0.320644	ITA
Sicilian	Sardinian	0.320644	ITA
Tuscan	Italian	0.338867	ITA
Trentino	Italian	0.338867	ITA

Umbrian	Italian	0.338867	ITA
Valle dAosta	Italian	0.338867	ITA
Venetian	Italian	0.338867	ITA
Lapp	Estonian	0.331338	EST
Liechtensteiner	Liechtenstein (country)	0.345662	LIE
Luxemburger	Luxemburg (country)	0.340903	LUX
Maltese	Italian	0.338867	ITA
Manx	Orcadian	0.340588	IMY
Monegasque	French	0.353319	FRA
Northern Irelander	UK (country)	0.345724	GBR
Norwegian	Orcadian	0.340588	NOR
Portuguese	Portugal (country)	0.345939	PRT
Azorean	.5 Italian, .5 French	0.346093	PRT
Madeiran	.5 Italian, .5 French	0.346093	PRT
Scottish	UK (country)	0.345724	GBR
Swedish	Sweden (country)	0.340435	SWE
Aland Islander	Orcadian	0.340588	FIN
Swiss	Switzerland (country)	0.342733	CHE
Suisse	Switzerland (country)	0.342733	CHE
Romansch	Italian	0.338867	ROM
Suisse Romane	Italian	0.338867	ROM
Welsh	UK (country)	0.345724	GBR
Scandinavian, Nordic	Orcadian	0.340588	SWE
Albanian	Albania (country)	0.323064	ALB
Azerbaijani	Uyghur	0.321912	AZE
Belourussian	Belarus (country)	0.321653	BLR
Bulgarian	Bulgaria (country)	0.318447	BGR
Carpathian	.5 Russian, .5 Italian	0.33026	ROM
Cossack	Russian	0.321653	RUS
Croatian	Croatia (country)	0.337452	HRV
Czechoslovakian	Czech Republic (country)	0.321794	CZE
Bohemian	Russian	0.321653	RUS
Estonian	Estonian	0.331338	EST
Livonian	Estonian	0.331338	EST
Finno Ugrian	Estonian	0.331338	FIN

Mordovian	Russian	0.321653	RUS
Voytak	Russian	0.321653	RUS
Georgian	Georgia (country)	0.315343	GEO
Germans from Russia	.5 French, .5 Orcadian	0.346954	DEU
Rom	.5 Russian, .5 Italian	0.33026	ROM
Hungarian	Estonian	0.331338	HUN
Magyar	Estonian	0.331338	HUN
Latvian	Latvia (country)	0.321653	LVA
Lithuanian	Lithuania (country)	0.321653	LTU
Macedonian	Macedonia (country)	0.329711	MKD
Ossetian	Balochi	0.311038	IRN
Polish	Poland (country)	0.321653	POL
Kashubian	Russian	0.321653	RUS
Romanian	Romania (country)	0.330442	ROM
Bessarabian	Estonian	0.331338	MDA
Moldavian	Moldava (country)	0.327403	MDA
Wallachian	.5 Russian, .5 Italian	0.33026	ROM
Russian	Russian	0.321653	RUS
Muscovite	Russian	0.321653	RUS
Serbian	Serbia (country)	0.330307	YUG
Slovak	Slovakia (country)	0.322838	SVK
Slovene	Slovenia (country)	0.330264	SVN
Sorb/Wend	Russian	0.321653	RUS
Bashkir	Yakut	0.315058	TUR
Chevash	Yakut	0.315058	TUR
Yakut	Yakut	0.315058	TUR
Tatar	Yakut	0.315058	TUR
Uzbek	Uyghur	0.321912	UZB
Ukrainian	Ukraine (country)	0.321678	UKR
Yugoslavian	Serbia (country)	0.330307	YUG
Slav	Russian	0.321653	RUS
Central European, nec	.5 Russian, .5 Italian	0.33026	ROM
Northern European, nec	Orcadian	0.340588	DNK
Southern European, nec	Italian	0.338867	ITA
Western European, nec	French	0.353319	FRA

Eastern European, nec	Russian	0.321653	RUS
European, nec	.5 Italian, .5 French	0.346093	DEU
Spaniard	Spain (country)	0.345652	ESP
Catalonian	Spain (country)	0.345652	ESP
Balearic Islander	Spain (country)	0.345652	ESP
Galician	.5 Italian, .5 French	0.346093	ESP
Mexican	Mexico (country)	0.292182	MEX
Mexican American	Mexico (country)	0.292182	MEX
Chicano/Chicana	Mexico (country)	0.292182	MEX
Nuevo Mexicano	Mexico (country)	0.292182	MEX
Californio	Mexico (country)	0.292182	MEX
Costa Rican	Costa Rica (country)	0.341711	CRI
Guatemalan	Guatemala (country)	0.273953	GTM
Honduran	Honduras (country)	0.302716	HND
Nicaraguan	Nicaragua (country)	0.310932	NIC
Panamanian	Panama (country)	0.296404	PAN
Salvadoran	El Salvador (country)	0.299874	SLV
Latin American	Mexico (country)	0.292182	MEX
Argentinean	Argentinia (country)	0.337511	ARG
Bolivian	Bolivia (country)	0.258775	BOL
Chilean	Chile (country)	0.283092	CHL
Colombian	Colombia (country)	0.306605	COL
Ecuadorian	Ecuador (country)	0.276056	ECU
Paraguayan	Paraguay (country)	0.28956	PRY
Peruvian	Peru (country)	0.264896	PER
Uruguayan	Uruguay (country)	0.34069	URY
Venezuelan	Venezuala (country)	0.304496	VEN
South American	Brazil (country)	0.324576	BRA
Puerto Rican	Dominican Republic (country)	0.322436	PRI
Cuban	Cuba (country)	0.331594	CUB
Dominican	Domincan Republic (country)	0.322436	DOM
Hispanic	Mexico (country)	0.292182	MEX
Spanish	.5 Italian, .5 French	0.346093	ESP
Spanish American	.5 Italian, .5 French	0.346093	ESP
Bahamian	Bahamas (country)	0.322089	BHS

Barbadian	Barbados (country)	0.319475	BRB
Belizean	Belize (country)	0.311487	BLZ
Bermudan	Bermuda (country)	0.329096	BMU
Cayman Islander	Jamaica (country)	0.32057	CYM
Jamaican	Jamaica (country)	0.32057	JAM
Dutch West Indies	Jamaica (country)	0.32057	ANT
Aruba Islander	Venezuala (country)	0.304496	ABW
St Maarten Islander	Antigua (country)	0.318978	ANT
Trinidadian/Tobagonian	Trinidad (country)	0.32142	TTO
Trinidadian	Trinidad (country)	0.32142	TTO
Tobagonian	Trinidad (country)	0.32142	TTO
U.S. Virgin Islander	Antigua (country)	0.318978	VIR
British Virgin Islander	Antigua (country)	0.318978	VIR
British West Indian	Bermuda (country)	0.329096	BMU
Turks and Caicos Islander	Bahamas (country)	0.322089	BHS
Anguilla Islander	Antigua (country)	0.318978	ATG
Dominica Islander	Dominca (country)	0.31651	DMA
Grenadian	Grenada (country)	0.318636	GRD
St Lucia Islander	St Kitts (country)	0.319547	LCA
French West Indies	St Kitts (country)	0.319547	KNA
Guadeloupe Islander	Dominca (country)	0.31651	DMA
Cayenne	Dominca (country)	0.31651	DMA
West Indian	Dominican Republic (country)	0.322436	DOM
Haitian	Haiti (country)	0.319228	HTI
Brazilian	Brazil (country)	0.324576	BRA
San Andres	Jamaica (country)	0.32057	JAM
Guyanese/British Guiana	Guyana (country)	0.313851	GUY
Providencia	Jamaica (country)	0.32057	JAM
Surinam/Dutch Guiana	Surinam (country)	0.320199	SUR
Algerian	Algeria (country)	0.332228	DZA
Egyptian	Egypt (country)	0.329321	EGY
Libyan	.	.	LBY
Moroccan	Morroco (country)	0.335216	MAR
Ifni	Morroco (country)	0.335216	MAR
Tunisian	Tunisia (country)	0.32949	TUN

North African	Egypt (country)	0.329321	EGY
Alhucemas	Morroco (country)	0.335216	MAR
Berber	Mozabite	0.34386	MAR
Rio de Oro	Morroco (country)	0.335216	MAR
Bahraini	Bahrain (country)	0.327284	BHR
Iranian	Iran (country)	0.314294	IRN
Iraqi	Iraq (country)	0.325699	IRQ
Israeli	.	.	ISR
Jordanian	Jordan (country)	0.328972	JOR
TransJordan	Jordan (country)	0.328972	JOR
Kuwaiti	Kuwait (country)	0.32845	KWT
Lebanese	Lebanon (country)	0.328391	LBN
Saudi Arabian	Saudi Arabia (country)	0.329846	SAU
Syrian	Syria (country)	0.327922	SYR
Armenian	Armenia (country)	0.317006	ARM
Turkish	Turkey (country)	0.315704	TUR
Yemeni	Oman (country)	0.327634	OMN
Omani	Oman (country)	0.327634	OMN
Muscat	Oman (country)	0.327634	OMN
Trucial Oman	Oman (country)	0.327634	OMN
Qatar	Qatar (country)	0.324068	QAT
Bedouin	Bedouin	0.334572	SAU
Kurdish	Balochi	0.311038	IRN
Kuria Muria Islander	Oman (country)	0.327634	OMN
Palestinian	Palestinian	0.329321	JOR
Gazan	Palestinian	0.329321	JOR
West Bank	Palestinian	0.329321	JOR
South Yemeni	Oman (country)	0.327634	YEM
Aden	Oman (country)	0.327634	YEM
United Arab Emirates	Saudi Arabia (country)	0.329846	ARE
Assyrian/Chaldean/Syriac	Syria (country)	0.327922	SYR
Middle Eastern	Saudi Arabia (country)	0.329846	SAU
Arab	Palestinian	0.329321	SAU
Angolan	Angola (country)	0.329822	AGO
Benin	Benin (country)	0.311931	BEN

Botswana	Botswana (country)	0.325655	BWA
Burundian	Burundi (country)	0.329497	BDI
Cameroonian	Cameroon (country)	0.317818	CMR
Cape Verdean	Cape Verde (country)	0.328605	CPV
Chadian	.	.	TCD
Congolese	Congo (country)	0.329822	COG
Equatorial Guinea	Equatorial Guinea (country)	0.329238	GNQ
Corsico Islander	Equatorial Guinea (country)	0.329238	GNQ
Ethiopian	.	.	ETH
Eritrean	.	.	ERI
Gabonese	Gabon (country)	0.329822	GAB
Gambian	Gambia (country)	0.284383	GMB
Ghanian	Ghana (country)	0.309695	GHA
Guinean	Guinea (country)	0.284383	GIN
Guinea Bissau	Guinea Bissau (country)	0.284383	GNB
Ivory Coast	Ivory Coast (country)	0.305893	CIV
Kenyan	.	.	KEN
Lesotho	Lesotho (country)	0.329822	LSO
Liberian	Liberia (country)	0.296039	LBR
Madagascan	Madagascar (country)	0.300658	MDG
Malian	Mali (country)	0.295182	MLI
Namibian	Namibia (country)	0.320899	NAM
Niger	Niger (country)	0.30862	NER
Nigerian	Nigeria (country)	0.311891	NGA
Fulani	Mandenka	0.284383	GIN
Hausa	Mandenka	0.284383	NGA
Ibo	Yoruba	0.314909	NGA
Tiv	Yoruba	0.314909	NGA
Rwandan	Rwanda (country)	0.329505	RWA
Senegalese	Senegal (country)	0.284383	SEN
Sierra Leonean	Sierra Leone (country)	0.284383	SLE
Somalian	.	.	SOM
Swaziland	Swaziland (country)	0.33031	SWZ
South African	South Africa (country)	0.329926	ZAF
Union of South Africa	South Africa (country)	0.329926	ZAF

Afrikaner	Orcadian	0.340588	NLD
Zulu	Bantu	0.329822	ZAF
Sudanese	.	.	SDN
Fur	.	.	SDN
Tanzanian	Tanzania (country)	0.326463	TZA
Togo	Togo (country)	0.303878	TGO
Ugandan	Uganda (country)	0.329822	UGA
Zairian	DRC (country)	0.329822	ZAR
Zambian	Zambia (country)	0.329822	ZMB
Zimbabwean	Zimbabwe (country)	0.329572	ZWE
African Islands	Bantu	0.329822	MOZ
Central African	.	.	.
East African	.	.	.
West African	Yoruba	0.314909	NGA
African	Black	0.318553	AA
Afghan	Afghanistan (country)	0.329023	AFG
Baluchi	Balochi	0.311038	PAK
Pathan	Pashtun	0.330319	PAK
Bengali	Bangladesh (country)	0.323009	BGD
Bhutanese	Bhutan (country)	0.289036	BTN
Nepali	Nepal (country)	0.241703	NPL
Asian Indian	India (country)	0.320623	IND
Andaman Islander	India (country)	0.320623	IND
Andhra Pradesh	India (country)	0.320623	IND
Assamese	India (country)	0.320623	IND
Goanese	India (country)	0.320623	IND
Gujarati	India (country)	0.320623	IND
Karnatakan	India (country)	0.320623	IND
Keralan	India (country)	0.320623	IND
Maharashtran	India (country)	0.320623	IND
Madrasi	India (country)	0.320623	IND
Mysore	India (country)	0.320623	IND
Naga	India (country)	0.320623	IND
Pondicherry	India (country)	0.320623	IND
Punjabi	Pakistan (country)	0.324431	PAK

Tamil	Brahui	0.313751	LKA
Pakistani	Pakistan (country)	0.324431	PAK
Sri Lankan	Sri Lanka (country)	0.320953	LKA
Singhalese	Sri Lanka (country)	0.320953	LKA
Veddah	Sri Lanka (country)	0.320953	LKA
Maldivian	Sri Lanka (country)	0.320953	LKA
Burmese	Myanmar (country)	0.302644	MMR
Shan	Myanmar (country)	0.302644	MMR
Cambodian	Cambodia (country)	0.299148	KHM
Khmer	Cambodian	0.298463	KHM
Chinese	China (country)	0.320225	CHN
Cantonese	China (country)	0.320225	CHN
Manchurian	China (country)	0.320225	CHN
Mongolian	Mongolia (country)	0.315546	MNG
Tibetan	China (country)	0.320225	CHN
Hong Kong	China (country)	0.320225	CHN
Macao	China (country)	0.320225	CHN
Filipino	Phillipines (country)	0.298659	PHL
Indonesian	Indonesia (country)	0.298463	IDN
Japanese	Japan (country)	0.313769	JPN
Ryukyu Islander	Japan (country)	0.313769	JPN
Okinawan	Japan (country)	0.313769	JPN
Korean	South Korea (country)	0.329926	KOR
Laotian	Laos (country)	0.271095	LAO
Meo	Miao	0.321474	CHN
Hmong	She	0.275142	CHN
Malaysian	Malaysia (country)	0.306422	MYS
Singaporean	Singapore (country)	0.317352	SIN
Thai	Thailand (country)	0.278977	THA
Black Thai	Thailand (country)	0.278977	THA
Western Lao	Laos (country)	0.271095	LAO
Taiwanese	China (country)	0.320225	CHN
Vietnamese	Vietnam (country)	0.298078	VNM
Katu	Cambodian	0.298463	KHM
Mnong	She	0.275142	CHN

Montagnard	Vietnam (country)	0.298078	VNM
Indochinese	Vietnam (country)	0.298078	VNM
Eurasian	.	.	.
Asian	.	.	.
Australian	Australia (country)	0.343894	AUS
Tasmanian	.	.	.
New Zealander	New Zealand (country)	0.327982	NZL
Polynesian	.5 Cambodian, .5 Melanesian	0.254042	WSM
Maori	.5 Cambodian, .5 Melanesian	0.254042	WSM
Hawaiian	.5 Cambodian, .5 Melanesian	0.254042	WSM
Part Hawaiian	.5 Cambodian, .5 Melanesian	0.254042	WSM
Samoan	.5 Cambodian, .5 Melanesian	0.254042	WSM
Tongan	Tonga (country)	0.256664	TON
Tokelauan	.5 Cambodian, .5 Melanesian	0.254042	TON
Cook Islander	.5 Cambodian, .5 Melanesian	0.254042	TON
Tahitian	.5 Cambodian, .5 Melanesian	0.254042	TON
Niuean	.5 Cambodian, .5 Melanesian	0.254042	TON
Micronesian	Micronesia (country)	0.210421	FSM
Guamanian	.5 Cambodian, .5 Melanesian	0.254042	MNP
Chamorro Islander	.5 Cambodian, .5 Melanesian	0.254042	GUM
Saipanese	.5 Cambodian, .5 Melanesian	0.254042	MNP
Palauan	Palau (country)	0.272599	PLW
Marshall Islander	Marshall Islands (country)	0.210078	MHL
Kosraean	Nauru (country)	0.241703	MHL
Chuukese	Nauru (country)	0.241703	MHL
Yap Islander	Nauru (country)	0.241703	MHL
Caroline Islander	Nauru (country)	0.241703	MHL
Kiribatese	.5 Cambodian, .5 Melanesian	0.254042	KIR
Nauruan	Nauru (country)	0.241703	MHL
Melanesian Islander	Melanesian	0.209622	SLB
Fijian	Fiji (country)	0.260992	FJI
New Guinean	PNG (country)	0.234712	PNG
Papuan	PNG (country)	0.234712	PNG
Solomon Islander	SI (country)	0.211849	SLB
Vanuatuan	Vanuatu (country)	0.211002	VUT

Pacific Islander	.5 Cambodian, .5 Melanesian	0.254042	OC
Oceania	.5 Cambodian, .5 Melanesian	0.254042	OC
Afro-American	Black	0.318553	AA
American Indian (all tribes)	Pima	0.246333	AI
Aleut	Pima	0.246333	AI
Eskimo	Pima	0.246333	AI
White/Caucasian	.5 Italian, .5 French	0.346093	WH
Greenlander	.	.	.
Canadian	Canada (country)	0.350252	CAN
Newfoundland	Canada (country)	0.350252	CAN
Nova Scotian	Canada (country)	0.350252	CAN
French Canadian	French	0.353319	FRA
Acadian	French	0.353319	FRA
American	USA (country)	0.335237	USA
United States	USA (country)	0.335237	USA
North American	USA (country)	0.335237	USA
