# Supplementary material for: Heterogeneity in disease resistance and the impact of antibiotics in the US
Source: Econ Hum Biol. Author manuscript; Available in PMC 2023 Feb 28. (PMC9972546; doi:10.1016/j.ehb.2022.101155)
Supplement: Appendix A - 1 [file NIHMS1876438-supplement-Appendix_A_-_1.pdf]

Appendix to Cook and Fletcher 2021
Online Only, Not Intended for Publication

A1 Summary Statistics

Table A1
Summary Statistics
1932-1942

Variable	Obs.	Mean	Std Deviation	Min	Max
HLA Susceptibility (Homozygosity)	48	0.6634	0.0065	0.6544	0.6906
Mixed HLA Susceptibility (Homozygosity)	48	0.6505	0.0028	0.6457	0.6612
Outcomes					
Bacterial Mortality Rate	527	191.51	74.71	66.7	618.3
Pre-treatment	239	220.47	73.76	125.08	592.78
Post-treatment	288	167.48	66.64	66.7	618.3
Residual Mortality Rate	527	890.88	132.37	566.19	1965.47
Pre-treatment	239	888.82	146.35	566.19	1965.47
Post-treatment	288	892.58	119.78	629.90	1212.9
Average Age,16-65	528	34.19	2.18	29.13	37.67
Pre-treatment	240	33.85	2.25	29.13	37.17
Post-treatment	288	34.47	2.07	29.92	37.67
(Individual by Birth Year) Years of Schooling	3,383,021	13.35	3.07	0	22
Pre-treatment	1,433,479	13.09	3.19	0	22
Post-treatment	1632	887.66	118.08	0	22
Time Varying Controls					
Annual Average Monthly Temperature (Fahrenheit)	527	51.76	8.00	37.86	70.96
Annual Average Monthly Precipitation (inches)	527	2.95	1.17	0.62	6.06
Pre-period (c.1936) State-Level Controls					
Demographic					
Frac. Black	48	9.40	13.37	0.06	49.8
Urbanization Rate	48	46.55	19.25	18.06	92.08
Frac. Foreign Born	48	9.05	6.87	0.4	24.66
Ethnic Diversity	48	0.87	0.03	0.78	0.92
Initial Population (in 1000s)	48	2621.87	2619.86	98.2	13247.2
In-migrants (in 1000s, c.1935-1940)	48	132.79	136.71	18.97	876.83
Out-migrants (in 1000s, c.1935-1940)	48	133.26	93.17	13.13	417.55
Infrastructure					
Schools per square mile	48	0.12	0.09	0.0029	0.36
Hospitals per square mile	48	0.0045	0.0068	0.0002	0.0334
Physicians per capita	48	0.0012	0.0003	0.0007	0.0018
Initial Real Income (in $1000s)	48	12.76	3.70	5.69	20.88
Fraction European Ancestry	48	0.8676	0.1364	0.4891	0.9876

Summary & Notes: This table provides summary statistics for all variables used in our analysis. Definitions and sources of each variable are given in Section A2. Arizona is the most HLA susceptible state, and Maine is the least.

Table A2

Additional Summary Statistics: Individual Census Controls

1932-1942

Variable	Obs.	Mean	Std Deviation	Min	Max
Race					
White	3,383,021	0.8833	0.3211	0	1
Black	3,383,021	0.0990	0.2987	0	1
American Indian	3,383,021	0.0071	0.0838	0	1
Chinese	3,383,021	0.0005	0.0230	0	1
Japanese	3,383,021	0.0009	0.0301	0	1
Other Asian or Pacific Islander	3,383,021	0.0005	0.0230	0	1
Other race	3,383,021	0.0058	0.0762	0	1
Two major races	3,383,021	0.0027	0.0516	0	1
Three or more major races	3,383,021	0.0002	0.0125	0	1
Sex					
Male	3,383,021	0.4848	0.50	0	1
Female	3,383,021	0.5152	0.50	0	1
Rural	3,383,021	0.3617	0.4804	0	1
Age†	3,383,021	52.18	9.03	37	68

† Fixed effects, or indicators for each age, used in estimation.

A2 Variable Descriptions and Sources

HLA Regressors of Interest

- HLA Susceptibility: This variable is a weighted average of state-level ancestral HLA homozygosity for individuals born between 1932 and 1936 (individuals born up to 5 years before the introduction of sulfa drugs in 1937).[1] Self-reported ancestry from the 5% sample of the 1980, 1990, and 2000 census are matched to country/ethnic HLA heterozygosity measures from Cook (2015). This matching is listed in the separate Matching Appendix.

  Individuals in the Census can report up to 2 ethnicities/ancestries. For those reporting 2 ancestries, we simply take the average of the matched HLA similarity score.

- Mixed HLA Susceptibility: Our primary way of calculating state-level HLA susceptibility takes the weighted average of each reported ethnicity's HLA homozygosity. This method assumes no mixing among different ethnic groups. The other extreme considers fully admixed populations. To account for this extreme, we take the weighted average of genetic variants (or alleles) to find the frequency of the variant in the larger (mixed) population. Expected homozygosity is then calculated using the admixed allele frequencies, creating a measure of HLA susceptibility for fully integrated populations.

  State-level allele frequencies are found in a similar manner as the base/segregated measure of HLA diversity: we simply match reported ancestry for those born 5 years prior to the 1937 intervention, to ethnic allele frequencies of Cook (2015). We then take the weighted average of these frequencies to create a state-level allele frequency. Mixed HLA susceptibility is then expected homozygosity calculated from these state-level allele frequencies.

Outcomes

- Bacterial Mortality Rate: The sum (excluding missing) of mortality rates (deaths per 100,000) from typhoid, scarlet fever, pertussis, tuberculosis, diphtheria, influenza and pneumonia, diarrhea and enteritis, maternal mortality, and syphilis. Data are given at the state-year level. The availability of data differs by year. Table 2 lists the time range for when each disease is listed in the National Vital Statistics Reports. Data from 1900-1930 are from Grant Miller's NBER dataset. Data from 1931-2000 have been digitized from the annual National Vital Statistics Reports.

  To maximize data, we use an unbalanced state panel. States enter the panel in the following years:

[1] Expected homozygosity is one minus expected heterozygosity, which is used in Cook (2015) and Ashraf and Galor (2013).

Initial Year:	State:
1900	CT, IN, ME, MA, MI, NH, NJ, NY, RI, VT
1906	PA
1908	CA, WA, WI
1909	OH
1910	CO, MD, MT, NC, UT
1911	MN, MO
1913	VA
1914	KS
1916	SC
1917	KY
1918	OR, TN
1919	DE, FL, MS
1920	IL, NE
1922	GA, ID, WY
1923	IA
1924	ND
1926	AZ, WV
1927	AR, LA
1928	AL, OK
1929	NV, NM
1930	SD
1933	TX

- Residual Mortality Rate: The total mortality rate (per 100,000) minus the bacterial mortality rate. Data are from the National Vital Statistics.
- Average Age, 16-65: The annual average age of a state's population, limited to those between 16 and 65 years of age. Data are by decade prior to 1930. These data are from age-bands in the decennial census and tabulated by Turner et al. (2007).
- Years of Schooling: An individual's reported years of schooling. Data are from the detailed educational attainment (EDUCD) of the 5% census samples for years 1980, 1990, and 2000 (Ruggles et al. 2020).

Time Varying Controls

- Annual Average Monthly Temperature: The annual average of monthly average temperature (in Fahrenheit). These data are from `https://www7.ncdc.noaa.gov/CDO/CDODivisionalSelect.jsp`.
- Annual Average Monthly Precipitation: The annual average of monthly average precipitation (in inches). These data are from `https://www7.ncdc.noaa.gov/CDO/CDODivisionalSelect.jsp`.

Demographic Controls

- Fraction Black: The 1936 state-level fraction of the population that is black. This data is by way of Lleras-Muney (2002, 2005).

- Urbanization Rate: The 1936 state-level urban fraction of the population. This data is by way of Lleras-Muney (2002, 2005).
- Fraction of Foreign Born: The 1936 state-level fraction of the population that is not native to the United States. This data is by way of Lleras-Muney (2002, 2005).
- Ethnic Fractionalization: Ethnic fractionalization is measured as a Hirfendahl index for the fraction of a state's population attributed to each reported ancestry/ethnicity for the base sample of Census respondents used to measure HLA diversity.
- In and Out Migrants, 1935-1940: The total number of migrants that moved into or out of a state from 1935-1940. Data are from the 1940 Census volume on internal migration (U.S. Bureau of the Census, 1943).
- Initial (c.1936) Population: The 1936 state population. Data from Turner et al. (2007).

Infrastructure Controls

- Schools per square mile: The 1936 number of schools per square mile. This data is by way of Lleras-Muney (2002, 2005).
- Hospitals per Square Mile: The 1936 number of hospitals per square mile. This data is by way of Lleras-Muney (2002, 2005).
- Education Expenditures per Capita: The 1936 state-level average education expenditure per capita. This data is by way of Lleras-Muney (2002, 2005).
- Physicians per Capita: The 1936 number of physicians per capita. This data is by way of Lleras-Muney (2002, 2005).
- Initial (c.1936) Income: The 1936 state-level real income. Data from Turner et al. (2007).

Additional Census Demographic Controls

- Race: An individual's reported race. From the RACE variable of the 5% census samples for years 1980, 1990, and 2000 (Ruggles et al. 2020).
- Sex: An individual's reported sex. From the SEX variable of the 5% census samples for years 1980, 1990, and 2000 (Ruggles et al. 2020).
- Rural: An indicator denoting whether an individual does not live in a metro area. From the METAREA variable of the 5% census samples for years 1980, 1990, and 2000 (Ruggles et al. 2020).
- Age: An individual's reported age, limited to those 30 and older. From the AGE variable of the 5% census samples for years 1980, 1990, and 2000 (Ruggles et al. 2020).

A3 Correlation of HLA Susceptibility and Ancestral Origins

Table A3

HLA Susceptibility and Continental Ancestry

	Std. HLA Susceptibility	Europe	Africa	Americas	Asia
Frac. ancestral to Europe	-0.8593<br>(0.0000)				
Frac. ancestral to Africa	0.3548<br>(0.0133)	-0.7410<br>(0.0000)			
Frac. ancestral to Americas	0.8501<br>(0.0000)	-0.5872<br>(0.0000)	-0.1065<br>(0.4712)		
Frac. ancestral to Asia	-0.1048<br>(0.9203)	0.0478<br>(0.7468)	-0.2335<br>(0.1101)	0.1463<br>(0.3211)	

Summary & Notes: This table gives the pair-wise correlations between state-level HLA susceptibility and the ancestral fraction from each continent. Both HLA susceptibility and continental ancestry are from the 5% sample of the 1980, 1990, and 2000 censuses.

Table A4

Mixed HLA Susceptibility and Continental Ancestry

	Std. Mixed HLA Susceptibility	Europe	Africa	Americas	Asia
Frac. ancestral to Europe	0.0716<br>(0.6288)				
Frac. ancestral to Africa	-0.5487<br>(0.001)	-0.7410<br>(0.0000)			
Frac. ancestral to Americas	0.5662<br>(0.0000)	-0.5872<br>(0.0000)	-0.1065<br>(0.4712)		
Frac. ancestral to Asia	0.0389<br>(0.7927)	0.0478<br>(0.7468)	-0.2335<br>(0.1101)	0.1463<br>(0.3211)	

Summary & Notes: This table gives the pair-wise correlations between state-level HLA susceptibility and the ancestral fraction from each continent. Both HLA susceptibility and continental ancestry are from the 5% sample of the 1980, 1990, and 2000 censuses.

A4 Additional Figures

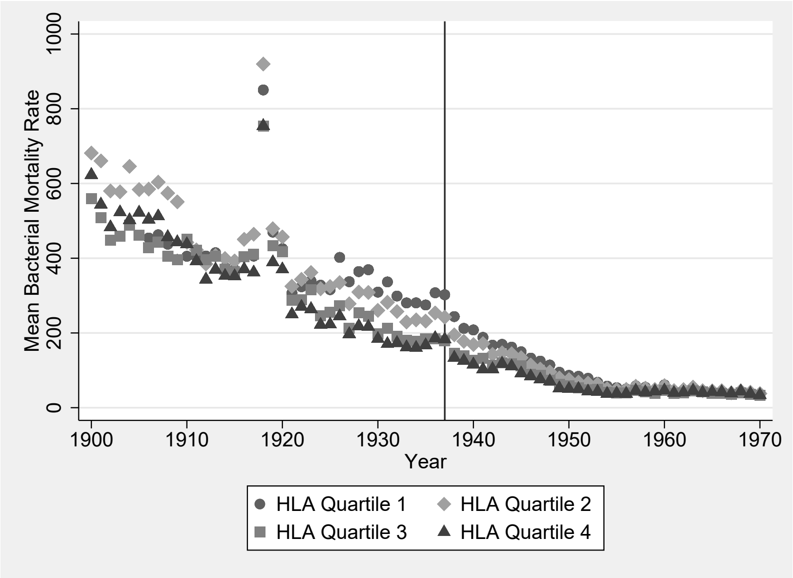

(a) Bacterial Mortality Rate

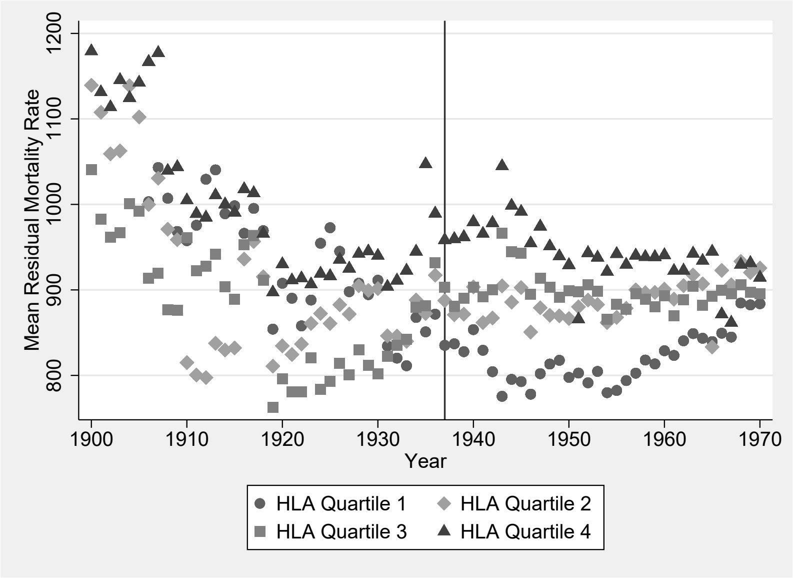

(b) Residual Mortality Rate

Figure A1

Mean Mortality Rate by HLA Quartiles, 1900-1970

Notes:

Sub-figure (a) plots the yearly average of bacterial mortality by quartiles of HLA susceptibility for a panel of states from 1900-1970. Sub-figure (b) plots a yearly average of all cause mortality rate less bacterial causes for HLA quartiles for 1900-1970.

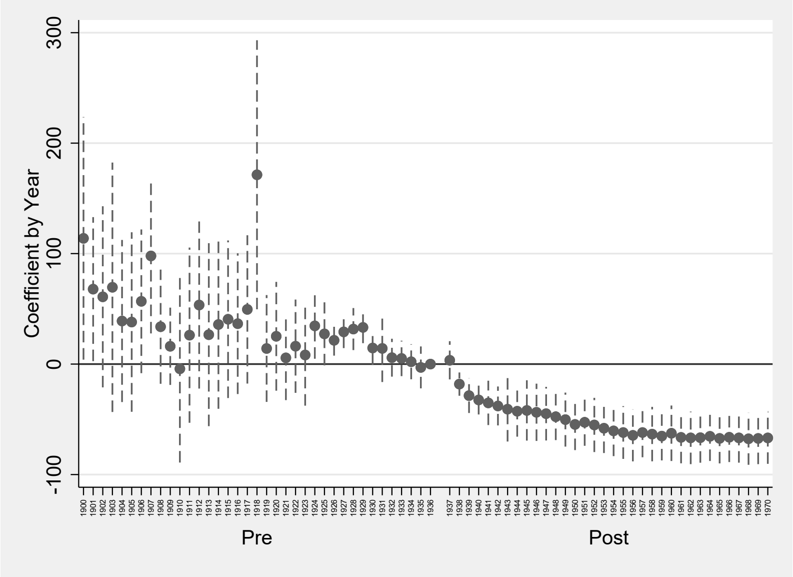

(a) Bacterial Mortality Rate

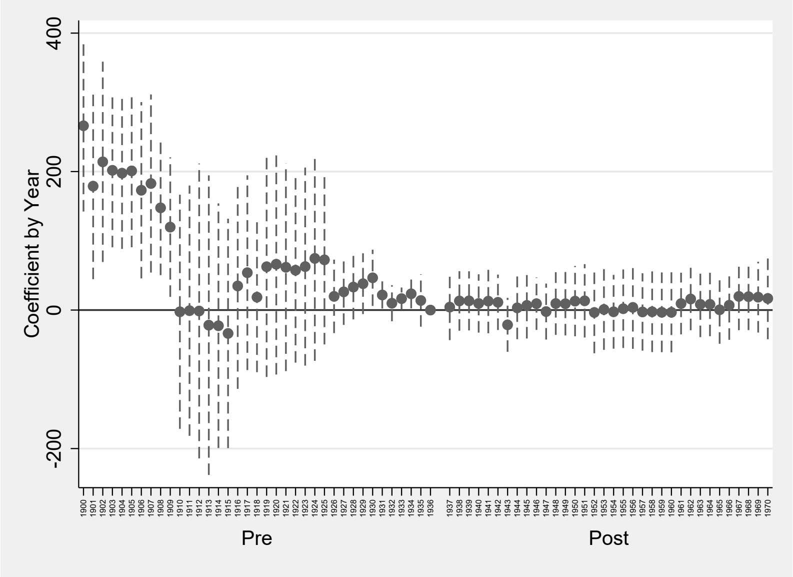

(b) Residual Mortality Rate

Figure A2
Effect of HLA Susceptibility by Year, 1900-1970

Notes:
This figure replicates the event figure given by Figure 2. The estimations follow that given by column (6) of Table 1, replacing the post-1937 indicator with annual indicators omitting 1936–the year prior to treatment. As seen in (a), there is a general relative increase in the coefficient of HLA susceptiblity in the early years of the 20th century, but after treatment in 1937 there is a clear decrease in the relative coefficient, suggesting a faster decline in the bacterial mortality rate for more exposed states. This differential decline is not seen for residual mortality in sub-figure (b). Of note, the sample of states is small (i.e., n=10) for the initial years, leading to noisy estimation. See the Variable Appendix for states by year.

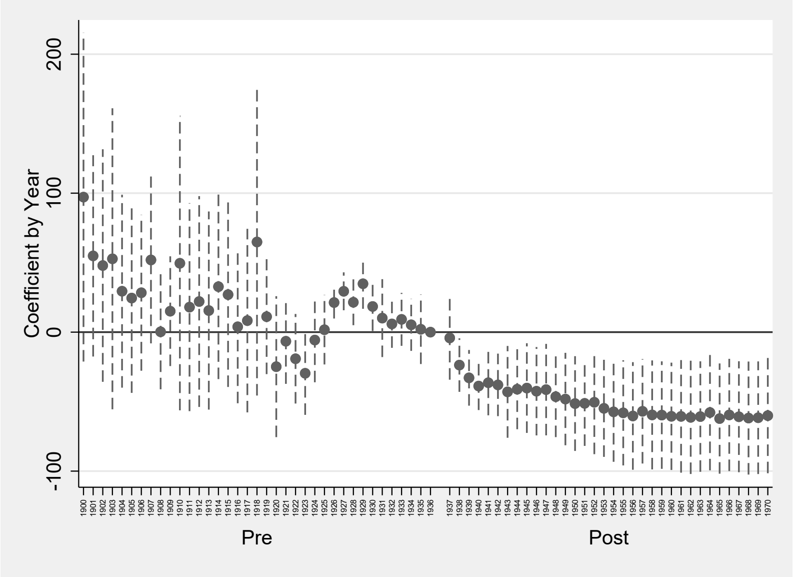

(a) Bacterial Mortality Rate

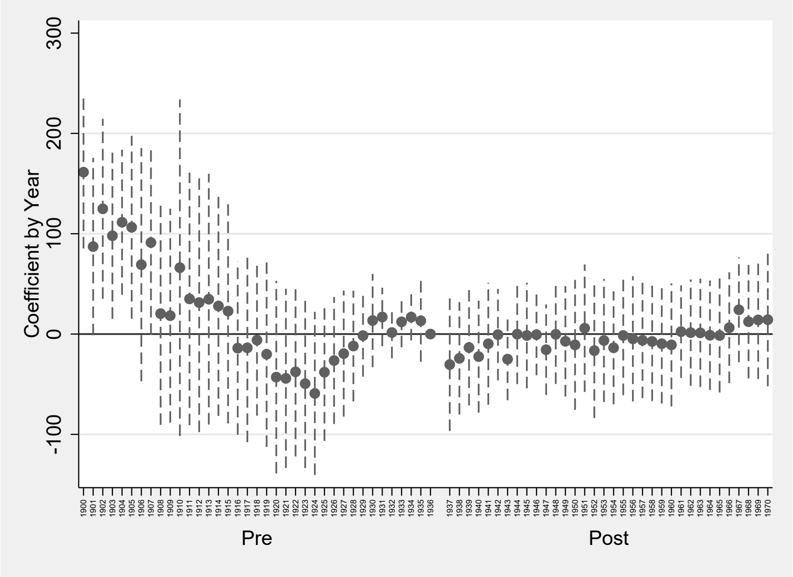

(b) Residual Mortality Rate

Figure A3
Effect of Mixed HLA Susceptibility by Year

Notes:
This figure replicates the event figure given by Figure 2. The estimations follow that given by column (6) of Table 2, replacing the post-1937 indicator with annual indicators omitting 1936–the year prior to treatment. As seen in (a), there is a general relative increase in the coefficient of HLA susceptiblity in the early years of the 20th century, but after treatment in 1937 there is a clear decrease in the relative coefficient, suggesting a faster decline in the bacterial mortality rate for more exposed states. This differential decline is not seen for residual mortality in sub-figure (b). Of note, the sample of states is small (i.e., n=10) for the initial years, leading to noisy estimation. See the Variable Appendix for states by year.

A5 Replicating Table 4 of Jayachandran et al. (2010)

Table A5
Replicating Table 4 of Jayachandran et al. (2010)

	Dependent variable: all mortality			
	(1)	(2)	(3)	(4)
Treated × Post-1937	596.5265***	448.4136***	251.8830***	-8.4236
	(38.5039)	(32.1555)	(31.5480)	(9.9190)
Treated × Post-1937 × Std. HLA Susc.	249.6813***	238.2671***	188.4743***	64.8882***
	(66.4467)	(54.1664)	(43.0618)	(8.0515)
Treated × Post-1937 × Trend	-16.4493***	-13.3681***	-8.7497***	-4.5137***
	(0.9902)	(0.8432)	(0.8017)	(0.1526)
Treated × Post-1937 × Trend × Std. HLA Susc.	-6.6787***	-6.5563***	-5.4025***	-1.5620***
	(1.7352)	(1.4603)	(1.1358)	(0.1764)
Marginal Effect for 1938 of Treated × Post-1937				
Std. HLA Susceptiblity = 0	-28.55***	-59.57***	-80.61***	-179.94***
	(2.57)	(3.42)	(4.53)	(10.23)
Std. HLA Susceptibility = 1	-32.66***	-70.44***	-97.43***	-174.41***
	(3.04)	(5.36)	(8.96)	(15.63)
Years	1932-1942	1925-1943	1925-1935, 1938-1943	1900-1970
Observations	1054	1762	1474	5482
R Sqr.	0.5749	0.4431	0.4688	0.7211

Notes:
This table replicates that of Jayachandran et al. (2010) while examining whether the post-1937 trend break differs by HLA susceptibility. This differential trend break is measured by the coefficient of Treated × Post-1937 × Trend × Std. HLA Susc., which is negative and statistically significant across differing sample periods, suggesting a greater post-1937 decline in more HLA susceptible states. Column (1) uses our base sample; column (2) uses the base sample from Jayachandran et al.; column (3) omits the years preceding treatment; and column (4) considers our long sample of 1900-1970. A Treated indicator and Treated× HLA susceptibility are included but not shown. State FE are also included. Standard errors are clustered by state with *, **, and *** being respectively associated with statistical significance at the 1, 5, and 10% levels.

A6 Post-1937 Linear Trend Break from HLA Susceptibility

Table A6

Baseline Panel Estimates: HLA Similarity

	Dependent variable: by panel					
	(1)	(2)	(3)	(4)	(5)	(6)
	Panel A. Bacterial Mortality Rate					
Post-1937 × Std. HLA Susceptibility	249.5577***	276.7145***	272.1183***	275.5738***	270.4062***	13.7288
	(67.0325)	(52.5989)	(53.6096)	(53.1120)	(55.1601)	(11.1590)
Post-1937 × Trend × Std. HLA Susceptibility	-6.6787***	-7.3359***	-7.3176***	-7.3294***	-7.2925***	-1.4921***
	(1.7504)	(1.3575)	(1.3716)	(1.3640)	(1.3858)	(0.2383)
Marginal Effect of Post-1937 × Std. HLA Susc.						
Year = 1938	-4.23	-2.05	-5.95**	-2.94	-6.71**	-42.97***
	(2.54)	(2.67)	(2.74)	(2.61)	(3.10)	(4.32)
Year = 1942	-30.95***	-31.39***	-35.22***	-32.26***	-35.88***	-48.94***
	(6.99)	(5.13)	(4.67)	(4.81)	(3.60)	(4.13)
Observations	527	527	527	527	527	2711
Years	1932-1942	1932-1942	1932-1942	1932-1942	1932-1942	1900-1970
	Panel B. Residual Mortality Rate					
Post-1937 × Std. HLA Similarity	24.8356	-64.1278	-65.8992	-60.0035	-58.9211	-26.4372
	(69.4242)	(42.7014)	(44.8198)	(43.2051)	(41.8089)	(25.4488)
Post-1937 × Trend × Std. HLA Susceptibility	-0.8757	1.2398	1.2103	1.2231	1.2257	0.1691
	(1.6752)	(1.1024)	(1.1136)	(1.1152)	(1.1148)	(0.5240)
Marginal Effect of Post-1937 × Std. HLA Susc.						
Year = 1938	-8.44	-17.02*	-19.91*	-13.53	-12.35	-20.01
	(9.83)	(10.15)	(10.93)	(10.66)	(9.41)	(18.24)
Year = 1942	-11.94	-12.06	-15.07	-8.64	-7.44	-19.33
	(8.42)	(11.23)	(11.35)	(11.75)	(11.08)	(18.60)
Observations	527	527	527	527	527	2711
Years	1932-1942	1932-1942	1932-1942	1932-1942	1932-1942	1900-1970
Controls:						
Fixed Effects:						
State	Y	Y	Y	Y	Y	Y
Year	Y					
Year × Census Division		Y	Y	Y	Y	Y
Time-varying:						
Mean Temperature		Y	Y	Y	Y	Y
Mean Precipitation		Y	Y	Y	Y	Y
Time-invariant × Post-1937						
Demographic Set			Y		Y	Y
Infrastructure Set				Y	Y	Y

Notes:
This table examines a differential trend break in our base DD analysis–i.e., with state and year (or year-by-census-division) FE and our full set of controls. In short, this table replicates Table 1 adding Post-1937 × Trend × Std. HLA Susceptibility to measure differences in the post-1937 linear trend tied to HLA susceptibility. As shown, this coefficient is negative and significant when regressing bacterial mortality suggesting that the estimated level decline in Table 1 is due to a differential linear trend tied to HLA susceptibility following treatment. No such difference is estimated for residual mortality in Panel B. Standard errors are clustered by state with *, **, and *** being respectively associated with statistical significance at the 1, 5, and 10% levels.

A7 Aggregate Years of Schooling

Table A7

Aggregate Cohort Effect on Schooling

	Dependent variable: Average Age, 16-65					
	(1)	(2)	(3)	(4)	(5)	(6)
	Panel A. Segregated HLA Susceptibility					
Post-1937 × Std. HLA	0.0925***	0.0821***	0.0986***	0.0779***	0.0871***	0.2811***
	(0.0154)	(0.0163)	(0.0189)	(0.0150)	(0.0167)	(0.0771)
Observations	528	528	528	528	528	2158
Years	1932-1942	1932-1942	1932-1942	1932-1942	1932-1942	1900-1970
Mean of Dependent Variable	34.19	34.19	34.19	34.19	34.19	34.38
Pre-period (Year<1937)	33.85	33.85	33.85	33.85	33.85	32.76
Post-period (Year≥1937)	34.47	34.47	34.47	34.47	34.47	34.91
	Panel B. Mixed HLA Susceptibility					
Post-1937 × Std. HLA	0.0060	0.0773***	0.1047***	0.0761***	0.0904***	0.2799***
	(0.0279)	(0.0232)	(0.0214)	(0.0198)	(0.0183)	(0.0799)
Observations	528	528	528	528	528	2158
Years	1932-1942	1932-1942	1932-1942	1932-1942	1932-1942	1900-1970
Mean of Dependent Variable	34.19	34.19	34.19	34.19	34.19	34.38
Pre-period (Year<1937)	33.85	33.85	33.85	33.85	33.85	32.76
Post-period (Year≥1937)	34.47	34.47	34.47	34.47	34.47	34.91
Controls:						
Fixed Effects:						
State	Y	Y	Y	Y	Y	Y
Year	Y					
Year × Census Division		Y	Y	Y	Y	Y
Time-varying:						
Mean Temperature		Y	Y	Y	Y	Y
Mean Precipitation		Y	Y	Y	Y	Y
Time-invariant × Post-1937						
Demographic Set			Y		Y	Y
Infrastructure Set				Y	Y	Y

Summary & Notes: This table replicates Table 5, replacing the individual panel with aggregate state panel. In so doing, we take the mean years of schooling by state of birth and birth year. Results are similar in magnitude and significance as the individual estimates. The demographic set of controls include the fraction of a state's population that is black in 1936, the fraction of a state's 1936 population that is foreign born, the urbanization rate in 1936, the state's population in 1936, the number of state-level in and out migrants between 1935-1940, and a measure of ethnic fractionalization based on the census-level reported ethnicity. The set of infrastructure controls includes education expenditures per capita in 1936, schools per square mile in 1936, hospitals per square mile in 1936, physicians per capita in 1936, and state-level real income in 1936. Standard errors are clustered by state. Statistical significance is denoted by *, **, and ***, representing significance at the 10, 5, and 1% levels, respectively.

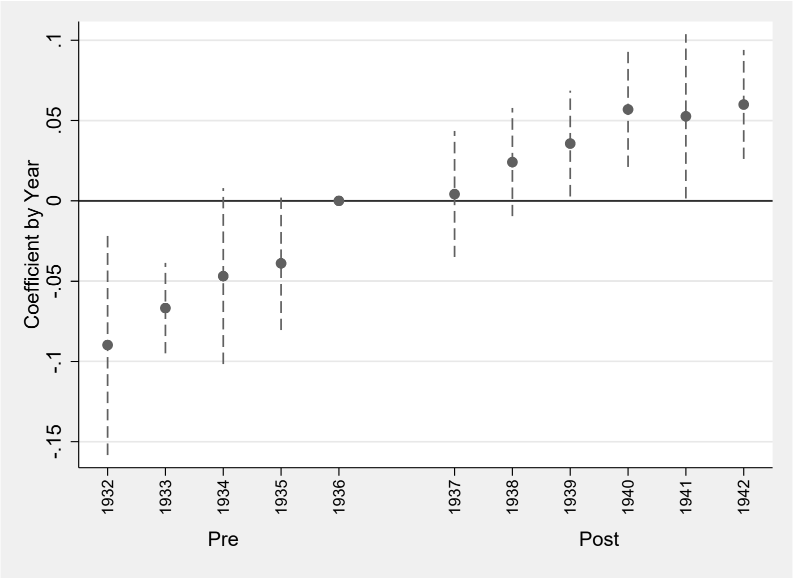

(a) Segregated HLA

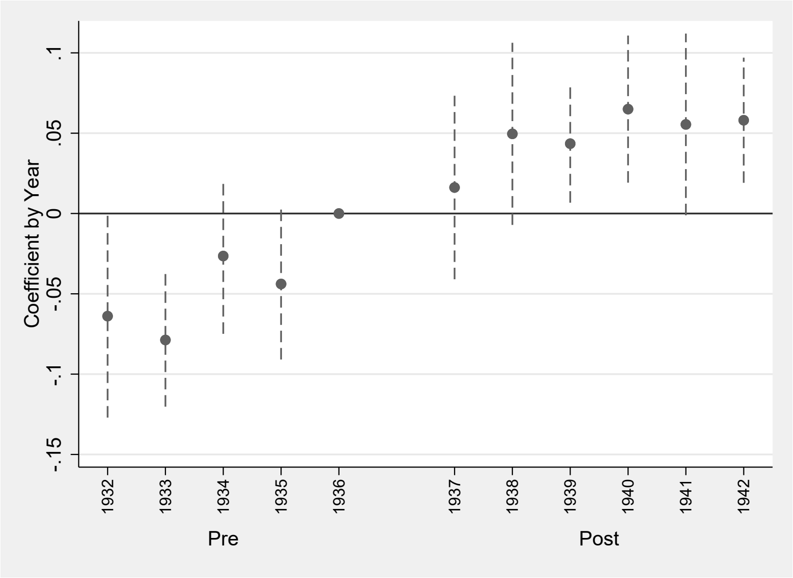

(b) Mixed HLA

Figure A4
Effect of HLA Susceptibility on Age of Labor Force by Year

Notes:
This figure plots annual coefficients relative to 1936 for the empirical specification of column (5) of Table A7. Sub-figure (a) plots the annual coefficient for the segregated measure of HLA susceptibility and shows positive effects on age following the introduction of sulfa drugs in 1937. As discussed in greater detail in the text, there is a pretrend for the segregated measure; however, we believe this is due spillover of the treatment into those early in childhood. Sub-figure (b) plots the annual coefficient for the mixed HLA susceptibility measure, showing very similar coefficients as those of the segregated measure.

A8 Effect on Income

Table A8
Contemporary Effect: Income

	Dependent variable: ln State Average Real Income					
	(1)	(2)	(3)	(4)	(5)	(6)
	Panel A. Segregated HLA Susceptibility					
Post-1937 × Std. HLA	0.0240***	0.0197***	0.0131	0.0158**	0.0171**	0.0160
	(0.0073)	(0.0064)	(0.0085)	(0.0060)	(0.0079)	(0.0235)
Observations	528	528	528	528	528	3408
Years	1932-1942	1932-1942	1932-1942	1932-1942	1932-1942	1900-1970
Mean of Dependent Variable	9.58	9.58	9.58	9.58	9.58	9.75
Pre-period (Year<1937)	9.40	9.40	9.40	9.40	9.40	9.40
Post-period (Year≥1937)	9.73	9.73	9.73	9.73	9.73	10.13
	Panel B. Mixed HLA Susceptibility					
Post-1937 × Std. HLA	0.0299**	0.0183	0.0104	0.0099	0.0123	0.0017
	(0.0116)	(0.0109)	(0.0094)	(0.0080)	(0.0100)	(0.0214)
Observations	528	528	528	528	528	3408
Years	1932-1942	1932-1942	1932-1942	1932-1942	1932-1942	1900-1970
Mean of Dependent Variable	9.58	9.58	9.58	9.58	9.58	9.75
Pre-period (Year<1937)	9.40	9.40	9.40	9.40	9.40	9.40
Post-period (Year≥1937)	9.73	9.73	9.73	9.73	9.73	10.13
Controls:						
Fixed Effects:						
State	Y	Y	Y	Y	Y	Y
Year	Y					
Year × Census Division		Y	Y	Y	Y	Y
Time-varying:						
Mean Temperature		Y	Y	Y	Y	Y
Mean Precipitation		Y	Y	Y	Y	Y
Time-invariant × Post-1937						
Demographic Set			Y		Y	Y
Infrastructure Set				Y	Y	Y

Summary & Notes: This table examines how the natural log of state-level average real income responded deferentially by HLA susceptibility to the introduction of sulfa drugs in 1937. Panel A uses a measure of HLA susceptibility that assumes fully segregated ancestral populations, and Panel B uses a measure of HLA susceptibility that assumes fully mixed ancestral populations. Contrary to other findings, we estimate roughly similar magnitudes for the segregated and mixed measures of HLA susceptibility. Estimates for the mixed measure, however, are imprecisely estimated and statistically insignificant different than zero. The demographic set of controls include the fraction of a state's population that is black in 1936, the fraction of a state's 1936 population that is foreign born, the urbanization rate in 1936, the state's population in 1936, the number of state-level in and out migrants between 1935-1940, and a measure of ethnic fractionalization based on the census-level reported ethnicity. The set of infrastructure controls includes education expenditures per capita in 1936, schools per square mile in 1936, hospitals per square mile in 1936, physicians per capita in 1936, and state-level real income in 1936. Standard errors are clustered by state. Statistical significance is denoted by *, **, and ***, representing significance at the 10, 5, and 1% levels, respectively.

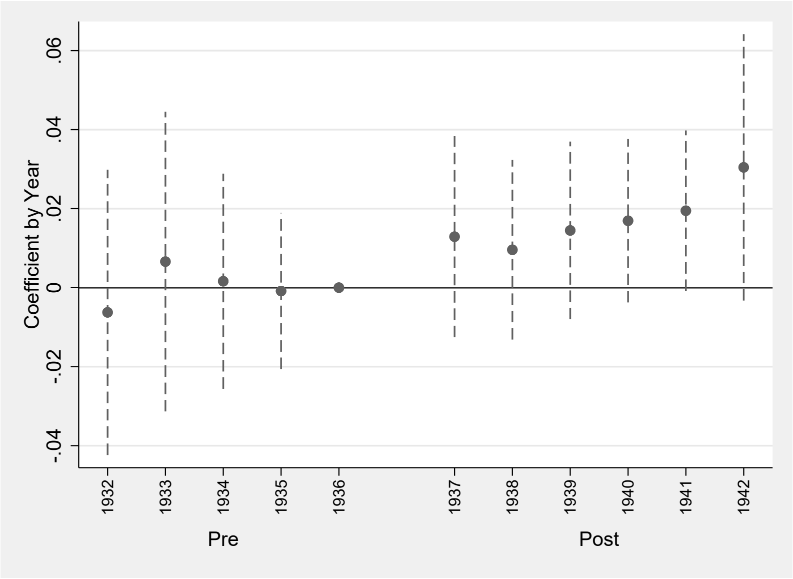

(a) Segregated HLA

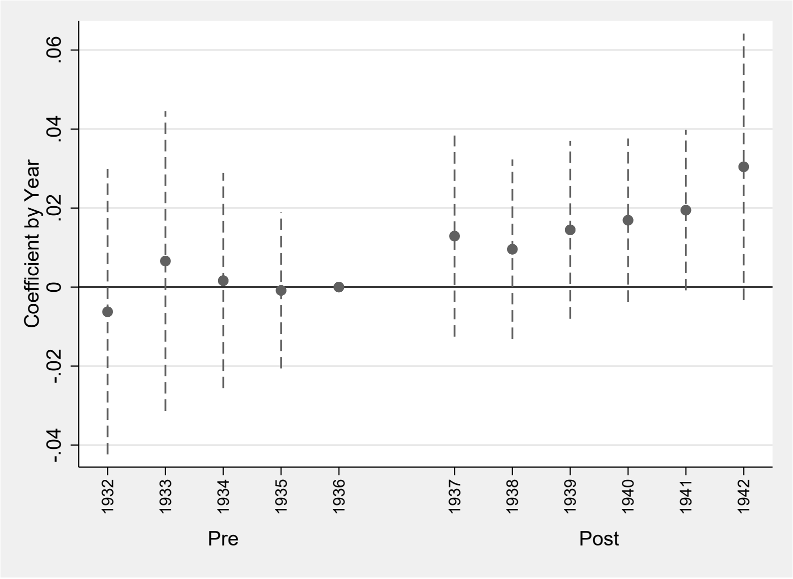

(b) Mixed HLA

Figure A5
Effect of HLA Susceptibility on Income by Year: Contemporary State Effect

Notes: Sub-figure (a) plots the annual coefficient (relative to 1936) of segregated HLA susceptibility on the natural log of average state real income. The specification follows that of column (5) of Table A8. Sub-figure (b) plots the annual coefficient of mixed HLA susceptibility; again following the specification of of column (5) of Table A8.

Table A9

Birth Cohort Effect: Income

	Dependent variable: ln Individual Real Income (age>30)					
	(1)	(2)	(3)	(4)	(5)	
	Panel A. Segregated HLA Susceptibility					
Post-1937 × Std. HLA	0.0079***	0.0107***	0.0113***	0.0097***	0.0110***	0.0180***
	(0.0018)	(0.0030)	(0.0026)	(0.0026)	(0.0028)	(0.0061)
Observations	3,372,657	3,372,657	3,372,657	3,372,657	3,372,657	18,179,156
Birth Years	1932-1942	1932-1942	1932-1942	1932-1942	1932-1942	1900-1970
Mean of Dependent Variable	8.86	8.86	8.86	8.86	8.86	8.97
Pre-period (Year<1937)	8.85	8.85	8.85	8.85	8.85	8.85
Post-period (Year≥1937)	8.86	8.86	8.86	8.86	8.86	9.06
	Panel B. Mixed HLA Susceptibility					
Post-1937 × Std. HLA	-0.0041	0.0058	0.0095***	0.0091***	0.0101***	0.0143**
	(0.0029)	(0.0035)	(0.0033)	(0.0031)	(0.0030)	(0.0054)
Observations	3,372,657	3,372,657	3,372,657	3,372,657	3,372,657	18,179,156
Birth Years	1932-1942	1932-1942	1932-1942	1932-1942	1932-1942	1900-1970
Mean of Dependent Variable	8.86	8.86	8.86	8.86	8.86	8.97
Pre-period (Year<1937)	8.85	8.85	8.85	8.85	8.85	8.85
Post-period (Year≥1937)	8.86	8.86	8.86	8.86	8.86	9.06
Controls:						
Fixed Effects:						
State	Y	Y	Y	Y	Y	Y
Year	Y					
Year × Census Division		Y	Y	Y	Y	Y
Time-varying:						
Mean Temperature		Y	Y	Y	Y	Y
Mean Precipitation		Y	Y	Y	Y	Y
Time-invariant × Post-1937						
Demographic Set			Y		Y	Y
Infrastructure Set				Y	Y	Y
Individual Set			Y		Y	Y

Summary & Notes: Instead of contemporary relationships, this table examines life long impacts arising from the 1937 treatment and its differential impact across states. To do so, we examine how the natural log of individual income (from the same 1980, 1990, and 2000 5% census samples used to calculate the HLA score) changes by birth cohort exposure to treatment. This table shows that individuals in states that were more exposed to infectious disease by their ancestral susceptibility also experienced relative gains in their income. Panel A uses a measure of HLA susceptibility that assumes fully segregated ancestral populations, and Panel B uses a measure of HLA susceptibility that assumes fully mixed ancestral populations. The demographic set of controls include the fraction of a state's population that is black in 1936, the fraction of a state's 1936 population that is foreign born, the urbanization rate in 1936, the state's population in 1936, the number of state-level in and out migrants between 1935-1940, and a measure of ethnic fractionalization based on the census-level reported ethnicity. The set of infrastructure controls includes education expenditures per capita in 1936, schools per square mile in 1936, hospitals per square mile in 1936, physicians per capita in 1936, and state-level real income in 1936. Individual controls include indicators for sex, urban/rural status, age, and race. We also include an indicator for those with no income in all estimations. Standard errors are clustered by state. Statistical significance is denoted by *, **, and ***, representing significance at the 10, 5, and 1% levels, respectively.

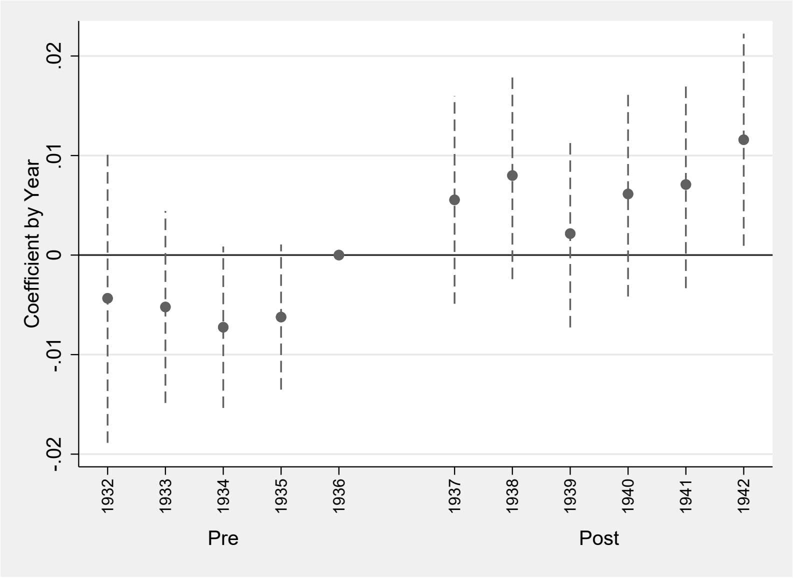

(a) Segregated HLA

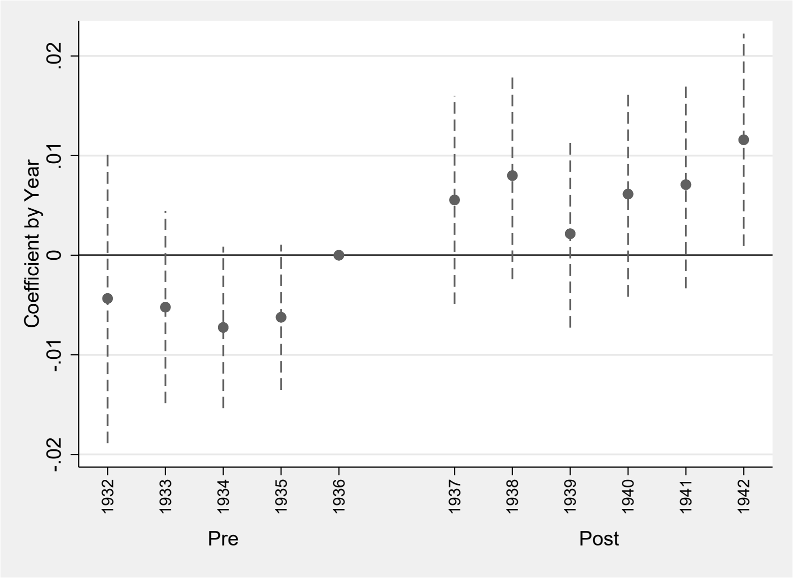

(b) Mixed HLA

Figure A6
Effect of HLA Susceptibility on Income by Year: Individual Cohort Effect

Notes: Sub-figure (a) plots the annual coefficient (relative to 1936) of segregated HLA susceptibility on the natural log of average state real income. The specification follows that of column (5) of Table A8. Sub-figure (b) plots the annual coefficient of mixed HLA susceptibility; again following the specification of of column (5) of Table A8.
